# Adjunctive therapies added to standard of care for diabetic foot ulcers: a systematic review and network meta-analysis with pairwise comparisons

**DOI:** 10.3389/fendo.2026.1891173

**Published:** 2026-07-31

**Authors:** Wenquan Luo, Ye Sun, Yujie Weng, Kai Huang, Yu Gan

**Affiliations:** 1 Tongde Hospital of Zhejiang Province Affiliated to Zhejiang Chinese Medical University (College of Integrated Traditional Chinese and Western Medicine Clinical Medicine), Hangzhou, China; 2 Department of Orthopedics, Tongde Hospital of Zhejiang Province Affiliated to Zhejiang Chinese Medical University, (Tongde Hospital of Zhejiang Province), Hangzhou, China; 3 Outpatient Comprehensive Service Center, Tongde Hospital of Zhejiang Province Affiliated to Zhejiang Chinese Medical University (Tongde Hospital of Zhejiang Province), Hangzhou, China

**Keywords:** adjunctive therapy, diabetic foot ulcer, network meta-analysis, standard of care, systematic review, wound healing

## Abstract

**Background:**

Diabetic foot ulcers (DFUs) remain one of the most disabling and costly complications of diabetes. Although standard of care (SOC) is the foundation of DFU management, many ulcers fail to heal adequately, prompting increasing use of adjunctive therapies. However, the comparative effectiveness of different adjunctive strategies remains uncertain.

**Objective:**

To evaluate and compare the efficacy and safety of adjunctive therapies added to SOC for patients with DFUs using systematic review, pairwise meta-analysis, and network meta-analysis.

**Methods:**

PubMed, Embase, CENTRAL, Web of Science, ClinicalTrials.gov, WHO ICTRP, and ChiCTR were searched from inception to February 18, 2026. Randomized controlled trials evaluating adjunctive therapies plus SOC for DFUs were included. Interventions were classified into eight clinically interpretable categories: oxygen-based therapies, device-based and physical wound therapies, growth factor and blood-derived therapies, placental and perinatal tissue-derived products, acellular matrix/skin substitute/scaffold products, cell-based and adipose-derived therapies, topical pharmacologic and bioactive agents, and nanotechnology-based, natural product, and bioactive dressing therapies. Outcomes included ulcer area reduction, complete healing, time to healing, adverse events, and amputation.

**Results:**

Eighty-four randomized controlled trials involving 7,232 participants were included. Compared with SOC alone, several adjunctive therapies improved healing-related outcomes. Device-based therapies, placental products, growth factor/blood-derived therapies, matrix/skin substitute products, and oxygen-based therapies were associated with greater ulcer area reduction. Oxygen-based therapies, matrix/skin substitute products, device-based therapies, placental products, topical pharmacologic/bioactive agents, and growth factor/blood-derived therapies increased the likelihood of complete healing. Cell-based/adipose-derived therapies, growth factor/blood-derived therapies, and matrix/skin substitute products shortened time to healing. Most adjunctive therapies did not increase adverse events, while placental products were associated with a lower risk of adverse events. Oxygen-based and growth factor/blood-derived therapies showed potential reductions in amputation risk, although the evidence was limited.

**Conclusions:**

Adjunctive therapies added to SOC may improve healing outcomes in patients with DFUs, but their benefits vary across clinical endpoints. Oxygen-based therapies, device-based therapies, placental products, growth factor/blood-derived therapies, and matrix/skin substitute products showed the most consistent potential benefits. Nevertheless, heterogeneity, sparse networks, variable certainty of evidence, and limited direct comparisons preclude a definitive treatment hierarchy. High-quality randomized trials with standardized outcome definitions and adequate follow-up are needed to clarify the optimal use of adjunctive therapies in DFU management.

**Systematic Review Registration:**

https://www.crd.york.ac.uk/PROSPERO/, identifier CRD420261385489.

## 1 Background

Diabetic foot ulceration (DFU) is one of the most common and clinically challenging chronic complications of diabetes (1).As the global prevalence of diabetes rises, DFU imposes an increasing burden on health-care systems. The International Diabetes Federation estimates that approximately 589 million adults worldwide are living with diabetes, with this number expected to increase further by 2050 (2).Previous studies suggest that 19% to 34% of people with diabetes will develop a foot ulcer during their lifetime (3), and that the global prevalence of DFU is approximately 6.3% (4). Beyond delayed wound healing, DFU is frequently complicated by infection, ischemia, neuropathy, and structural foot deformity, making it a major contributor to hospitalization, amputation, and reduced quality of life in this population (1).

Standard care for DFU typically includes wound debridement, offloading, infection control, glycaemic management, vascular assessment and revascularization when appropriate, moisture-retentive dressings, and multidisciplinary care (5). Although optimized standard care remains the foundation of DFU management, many ulcers heal slowly or deteriorate despite appropriate treatment (3). This has prompted interest in adjunctive therapies intended to improve healing when added to standard care (6). These include oxygen-based therapies, negative pressure and other device-based approaches, growth factors and bioactive agents, placental-derived products, skin substitutes and matrix-based materials, autologous blood-derived products, and other topical or systemic adjuncts (7). Because these interventions differ in mechanism, target population, cost, and feasibility, pooling them as a single treatment category may obscure clinically meaningful differences between therapeutic strategies (6).

Previous systematic reviews and meta-analyses of adjunctive therapies for DFU have generally focused on individual interventions, or have compared adjunctive treatments as a single broad category with standard care (8). Although these studies can indicate whether a specific treatment is likely to be beneficial, they provide limited information on how different adjunctive strategies compare with one another (9). Network meta-analysis can address this gap by integrating direct and indirect evidence within a single analytical framework, allowing several interventions to be compared simultaneously and offering a more useful basis for treatment selection (10). In DFU research, however, adjunctive interventions are highly diverse, many trials are small, comparators differ across studies, and outcome reporting is inconsistent (11). Classifying interventions too narrowly can lead to sparse or poorly connected networks, whereas overly broad grouping may weaken clinical interpretability. A clinically grounded reclassification of adjunctive therapies according to mechanism of action and practical use is therefore needed (6).

Accordingly, we classified adjunctive therapies for DFU into eight clinically interpretable categories and used network meta-analysis to compare their relative efficacy and safety, when added to standard care, for ulcer area reduction, complete wound healing, healing time, adverse events, and amputation (9). To improve interpretability and assess the consistency of the evidence, we also performed pairwise meta-analyses comparing each intervention category with standard care, with sensitivity analyses, subgroup analyses, and meta-regression used to explore potential sources of heterogeneity (12). By integrating network and pairwise meta-analytic approaches, this study provides a structured, clinically relevant synthesis to inform the selection of adjunctive therapies for DFU (13).

## 2 Materials and methods

This systematic review and network meta-analysis assessed the relative efficacy and safety of adjunctive therapies used alongside standard care for diabetic foot ulcers. The review was designed, conducted, and reported according to the PRISMA 2020 statement and the PRISMA extension for network meta-analyses (PRISMA-NMA) (9, 14). The protocol was prospectively registered in PROSPERO (International Prospective Register of Systematic Reviews; CRD420261385489). Treatment rankings and indirect comparisons were treated as exploratory and interpreted in light of the direct evidence, network structure, risk of bias, and certainty of evidence.

### 2.1 Data sources and search strategy

We searched PubMed, Embase, the Cochrane Central Register of Controlled Trials (CENTRAL), and the Web of Science Core Collection from inception to February 18, 2026. To capture unpublished and ongoing randomized controlled trials, we also searched ClinicalTrials.gov, the WHO International Clinical Trials Registry Platform (ICTRP), and the Chinese Clinical Trial Registry (ChiCTR). We manually screened the reference lists of eligible studies and relevant systematic reviews for additional records.

The search strategy was built around three concepts: diabetic foot ulcers, randomized controlled trials, and adjunctive therapies. Controlled vocabulary terms, including MeSH and Emtree, were combined with free-text terms and adapted for each database. Search terms for diabetic foot ulcers included “diabetic foot,” “diabetic foot ulcer,” and “diabetes-related foot ulcer”; trial-related terms included “randomized controlled trial,” “randomized,” “randomised,” “randomly,” “clinical trial,” and “placebo.” Adjunctive therapy terms covered oxygen-based treatments, negative-pressure wound therapy, physical therapies, growth factors and blood-derived products, biomaterials and extracellular matrix products, placenta-derived tissue products, cell-based therapies, topical pharmacologic and bioactive agents, natural products, and active dressings. No language restrictions were applied. The complete search strategy is provided in **Supplementary Table 2**.

### 2.2 Selection criteria

Eligibility criteria were prespecified according to the PICOS framework, including participants, interventions, comparators, outcomes, and study design.

#### 2.2.1 Inclusion criteria

##### 2.2.1.1 Participants

We included randomized studies of adults with diabetes and foot ulcers below the ankle. Diabetic foot ulcer was defined as a break in the skin of the foot extending at least through the epidermis into the dermis, with or without peripheral neuropathy, infection, or peripheral arterial disease. Studies of mixed chronic wound populations were eligible only when data for participants with diabetic foot ulcers were reported separately or could be extracted separately.

##### 2.2.1.2 Study design

Eligible designs were randomized controlled trials, including parallel-group and eligible multi-arm trials. When several reports described the same trial, the report with the largest sample size, longest or most complete follow-up, or most complete outcome reporting was used as the primary source; companion reports were consulted to supplement trial characteristics and outcome data.

##### 2.2.1.3 Interventions

We included adjunctive therapies for diabetic foot ulcers when administered in addition to standard of care (SOC). Based on mechanism of action, therapeutic modality, and mode of clinical use, interventions were grouped into eight categories: oxygen-based therapies (OXY); device-based and physical wound therapies (DEV); growth factor and blood-derived therapies (GFB); placental and perinatal tissue-derived products (PLA); acellular matrix, skin substitute, and scaffold products (MSS); cell-based and adipose-derived therapies (CAD); topical pharmacologic and bioactive agents (TBA); and nanotechnology-based, natural product, and bioactive dressing therapies (NND).

OXY comprised hyperbaric oxygen therapy (HBOT), topical oxygen therapy (TOT), and continuous diffusion oxygen therapy (CDO). DEV comprised negative-pressure wound therapy or vacuum-assisted closure (NPWT/VAC), extracorporeal shockwave therapy (ESWT), and ultrasound therapy. GFB comprised platelet-rich plasma or platelet-rich gel (PRP/PRG), platelet-derived growth factor or becaplermin (PDGF/becaplermin), and recombinant human epidermal growth factor (rhEGF). PLA comprised human amniotic membrane (HAM), dehydrated human amnion/chorion membrane (dHACM/HACM), human umbilical cord-derived products (HUCA/EpiCord), Grafix, and amniotic gel. MSS comprised acellular dermal matrix (ADM), Integra, bilayer matrices, extracellular matrix and other matrix products (ECM/matrix products), fish-skin grafts, glass-fiber matrices, and collagen gel. CAD comprised adipose-derived stem cells, lipoaspirate-derived cells, autologous keratinocytes, and fat grafting combined with PRP. TBA comprised topical erythropoietin, topical esmolol, ON101/macrophage-regulating therapy, ENERGI-F703, NorLeu3A, and P1G10. NND comprised zinc oxide nanoparticles, quercetin-oleic acid nanohydrogels, and other nanoformulated or naturally derived active dressings.

Because the broad categories included therapies that differ in mechanism, formulation, and delivery, this classification was chosen to balance clinical interpretability with network connectivity. It reduced sparsity in the treatment network but may have introduced within-category heterogeneity. Category-level pooled estimates should therefore be interpreted as average effects for a therapeutic class, not as definitive estimates for any individual product or technique. Where sufficient studies were available within a category, subgroup analyses were conducted to examine differences among specific therapeutic approaches.

##### 2.2.1.4 Comparators

Eligible comparators were standard of care (SOC) or another adjunctive therapy that met the inclusion criteria. SOC was defined as routine diabetic foot ulcer care, including wound assessment, debridement, simple dressings, offloading, infection control, glycaemic management, and, where clinically indicated, assessment of ischaemia and revascularisation.

In the network meta-analysis, SOC served as the common reference node. For trials comparing two adjunctive therapies, both groups had to receive the same, or broadly comparable, SOC so that the treatment contrast primarily reflected the adjunctive intervention itself. Placebo, sham treatment, or inactive dressings were treated as SOC comparators when both groups received SOC and the placebo or sham component was unlikely to have an active therapeutic effect. When the control group received another active adjunctive therapy, an enhanced dressing, or a device-based intervention, the comparison was included as a direct active-comparator contrast in the network. Trials with incompletely reported SOC were retained if the comparator was clearly described as routine diabetic foot ulcer care or usual care in the study setting; this limitation was considered in the assessment of risk of bias, indirectness, and heterogeneity. Multiple reports or follow-up phases from the same trial were deduplicated at the trial level to avoid counting the same participants more than once in the primary analysis of any given outcome.

##### 2.2.1.5 Outcomes

Studies were eligible if they reported at least one of the following outcomes: complete ulcer healing, reduction in ulcer area, time to healing, adverse events, or amputation. When multiple time points were reported for the same outcome, we preferentially extracted the prespecified primary endpoint, the end-of-treatment assessment, or the clinically conventional time point closest to 12 weeks. For safety and amputation outcomes, cumulative event counts, or events recorded over the longest available follow-up, were extracted preferentially. Variation in reporting windows across studies was considered a potential source of clinical heterogeneity when interpreting the findings.

#### 2.2.2 Exclusion criteria

Studies were excluded for any of the following reasons:

1. Duplicate reports from the same cohort or trial, including reports at different follow-up points, when participant-level overlap could not be reliably resolved.
2. Key outcome data were unavailable or insufficiently defined, and an effect estimate could not be derived through author contact or appropriate data conversion.
3. The study was not a randomized controlled trial, such as a non-randomized study, review, or case report.

All retrieved records were imported into reference management software and deduplicated. Two reviewers independently screened titles and abstracts, reviewed full texts, and assessed eligibility against the prespecified criteria. Disagreements were resolved through discussion, with unresolved discrepancies adjudicated by a third reviewer. Reasons for exclusion at the full-text stage were recorded, and the selection process was summarized in a PRISMA 2020 flow diagram.

### 2.3 Data extraction and quality assessment

Two reviewers independently extracted data using a standardized form that was piloted and calibrated before use. Extracted items included first author, publication year, country or region, sample size, available baseline characteristics (age, sex, ulcer grade, ulcer area, ulcer duration, and related variables), follow-up duration, details of the experimental and control interventions (dose, frequency, treatment duration, and whether the intervention was added to SOC), and raw outcome data.

For dichotomous outcomes, including complete ulcer healing, adverse events, and amputation, we extracted the number of events and the number of participants in each treatment group. Complete ulcer healing was defined as full epithelialization, complete wound closure, or complete healing as reported by the trial authors. Overall adverse events were extracted preferentially; when treatment-related or serious adverse events were reported separately, these were recorded and used in supplementary analyses. For amputation, we extracted the number of participants who underwent any lower-limb amputation.

For potentially duplicate publications or multiple reports from the same trial, we cross-checked the trial name, registration number, study centers, recruitment period, sample size, and intervention protocol. Reports based on the same participant population were counted only once for each outcome; companion reports were used only to supplement baseline characteristics, follow-up information, or safety data. Multi-arm trials were handled according to prespecified rules that preserved the within-trial correlation structure and avoided double-counting shared control groups.

For ulcer area reduction, we extracted the mean change from baseline and corresponding standard deviation. For time to healing, we preferentially extracted the mean time from randomization to complete healing and corresponding standard deviation, converting values to a common unit of weeks or days as appropriate. When only medians, interquartile ranges, or ranges were reported, means and standard deviations were estimated when methodologically appropriate. The standard deviation of change scores was derived from baseline and endpoint standard deviations and their correlation coefficient using the following formula:


$$SDc\text{hange}=\sqrt{{\text{Baseline SD}}^{2}+{\text{Endpoint SD}}^{2}-2R\cdot \text{Baseline SD}\cdot \text{Endpoint SD}}$$

When the correlation coefficient could not be derived from the study itself, R was imputed from the trial with the largest sample size, the lowest risk of bias, and available change-score data. For dichotomous outcomes, event counts and group totals were extracted for each arm.

### 2.4 Risk-of-bias assessment

Risk of bias in the included randomized controlled trials was assessed with the Cochrane Risk of Bias 2 tool (RoB 2). RoB 2 evaluates outcome-specific bias across five domains: the randomization process, deviations from intended interventions, missing outcome data, measurement of the outcome, and selection of the reported result. Each domain was rated as low risk, some concerns, or high risk according to the RoB 2 signalling questions, and an overall risk-of-bias judgement was assigned for each outcome.

Two reviewers independently completed the assessments. Disagreements were resolved by discussion or, when required, adjudication by a third reviewer. Because outcomes in diabetic foot ulcer trials, including complete healing, change in ulcer area, time to healing, adverse events, and amputation, may differ in their susceptibility to bias from lack of blinding, incomplete follow-up, and outcome ascertainment, risk of bias was interpreted at the outcome level rather than reduced to a single study-level quality score. The risk-of-bias assessments informed the certainty-of-evidence evaluation and were summarized graphically.

### 2.5 Statistical analysis

Analyses were conducted in Stata 17.0 MP. We first performed a network meta-analysis to compare the relative efficacy and safety of adjunctive therapy categories when added to standard of care (SOC). Continuous outcomes were expressed as standardized mean differences (SMDs) with 95% confidence intervals (CIs), and dichotomous outcomes as odds ratios (ORs) with 95% CIs. For continuous outcomes with opposite clinical directions, effect estimates were aligned before pooling, such that positive values indicated greater reduction in ulcer area and negative values indicated shorter time to healing. Multi-arm trials were modelled while preserving within-study correlations to avoid double-counting shared control groups. For dichotomous outcomes with zero events or zero non-events, continuity correction was applied; rare outcomes, including adverse events and amputation, were interpreted with caution.

The network meta-analysis used a random-effects model under the consistency assumption. Between-study variance (τ²) was estimated by restricted maximum likelihood (REML). Network geometry was shown in network plots, with node size proportional to the total sample size for each treatment category and edge thickness proportional to the number of studies contributing direct comparisons. Transitivity was assessed by comparing potential effect modifiers across treatment comparisons, including baseline ulcer area, ulcer severity, infection or ischaemia status, follow-up duration, SOC components, adherence to offloading, intervention intensity, and comparator type. In networks containing closed loops, inconsistency between direct and indirect evidence was assessed using a global inconsistency test, node-splitting, and loop-specific inconsistency factors (IFs); p<0.10 was considered a potential signal of inconsistency. Treatment rankings were summarized using the surface under the cumulative ranking curve (SUCRA), probability of being the best treatment, and mean rank. Rankings were treated as exploratory and interpreted together with effect estimates, 95% CIs, the amount of direct evidence, network structure, and certainty of evidence.

To complement the network meta-analysis and examine robustness, we conducted conventional pairwise meta-analyses comparing each adjunctive therapy category plus SOC with SOC alone for ulcer area reduction, complete ulcer healing, time to healing, adverse events, and amputation. Pairwise analyses used the same effect measures as the network meta-analysis. Heterogeneity was assessed using the I² statistic and χ² test. Fixed-effect models were used when I² was ≤50% and clinical heterogeneity was limited; random-effects models were used when I² was >50% or when clear clinical heterogeneity was present. For comparisons with substantial heterogeneity or few studies, pooled estimates were interpreted primarily as exploratory direct evidence. Robustness was assessed using leave-one-out sensitivity analyses. Subgroup analyses by specific intervention or treatment regimen were conducted to explore within-category differences. Meta-regression with study-level follow-up duration as a covariate was used to explore sources of heterogeneity; given the small number of studies in several comparisons, these analyses were considered exploratory. When enough studies were available, funnel plots or comparison-adjusted funnel plots were used to assess publication bias and small-study effects. When study numbers were insufficient, funnel plots were not used as the primary basis for judging publication bias.

### 2.6 Certainty of evidence

The certainty of evidence for the network meta-analysis was assessed using the GRADE framework and CINeMA (Confidence in Network Meta-Analysis). Evidence from randomized controlled trials was initially rated as high certainty. Certainty was judged across six domains: within-study bias, indirectness, imprecision, heterogeneity, incoherence, and reporting bias, including publication bias and small-study effects.

Within-study bias was assessed using RoB 2.0 and summarized at the comparison level through the CINeMA contribution matrix, weighted according to each study’s contribution to the network estimate. Indirectness was assessed against the assumptions of transitivity and exchangeability. Prespecified potential effect modifiers included baseline ulcer area, infection or ischaemia status, ulcer severity, components of SOC, follow-up duration, and intervention intensity. We assessed whether direct and indirect evidence were sufficiently comparable with respect to participants, interventions, comparators, and outcome measurement.

Imprecision was judged against prespecified minimum clinically important difference (MCID) thresholds: an SMD of 0.5 for continuous outcomes and an OR of 1.25 for dichotomous outcomes. Judgements also considered whether the 95% CI crossed the line of no effect or a clinically important threshold. Heterogeneity was assessed using τ², prediction intervals, and clinical heterogeneity. In networks with closed loops, incoherence was evaluated using node-splitting/SIDE and the design-by-treatment interaction model. Reporting bias was assessed by considering trial registration, grey-literature searches, and small-study effects suggested by comparison-adjusted funnel plots. Each domain was rated as no concerns, some concerns, or major concerns and downgraded according to GRADE principles. Overall certainty was classified as high, moderate, low, or very low.

## 3 Results

### 3.1 Study selection and characteristics

**Figure 1:**
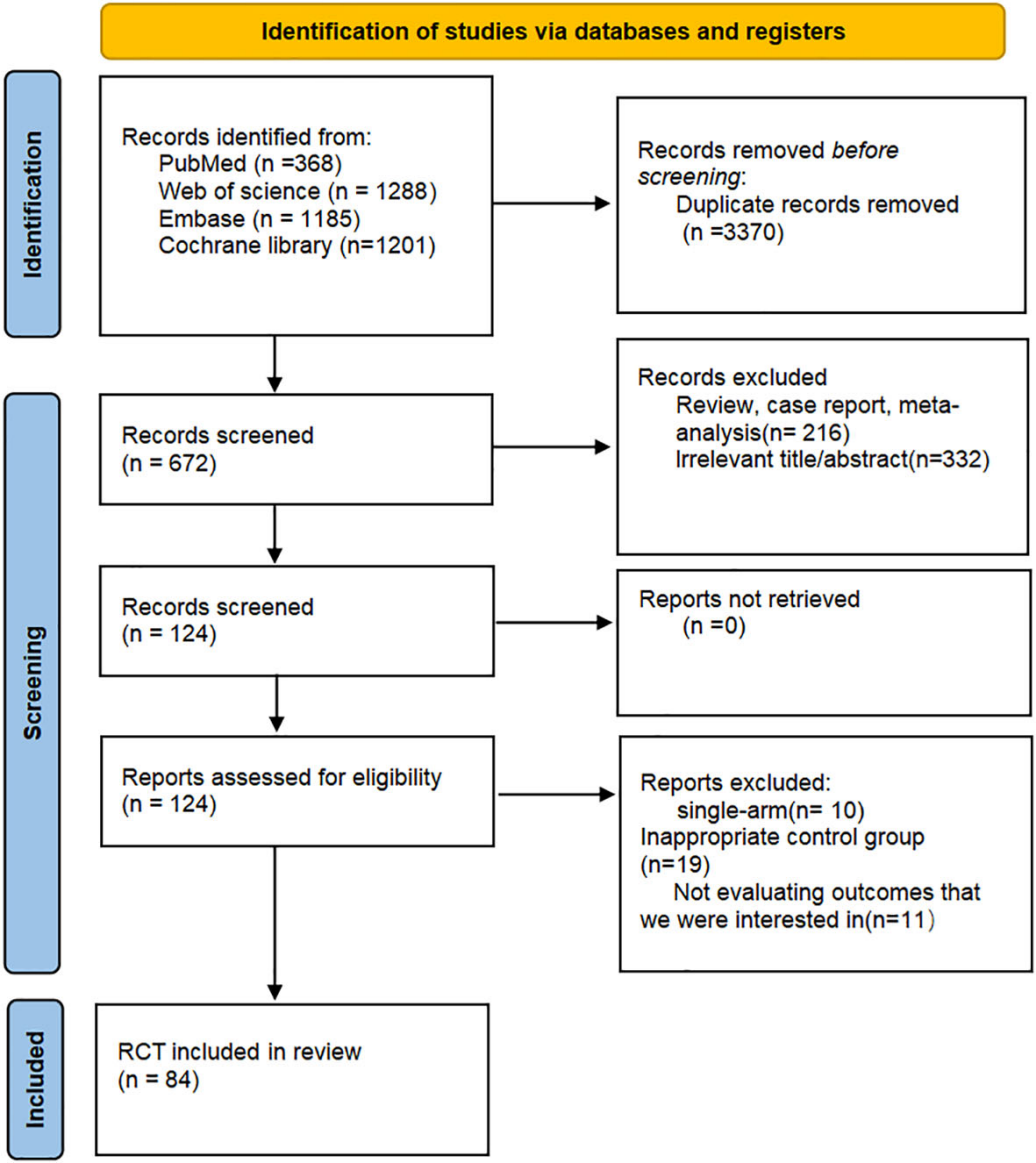
PRISMA flow diagram of study selection.

The database searches identified 4,042 records. After deduplication and title–abstract screening, 124 reports were reviewed in full text; 84 studies met the eligibility criteria and were included in the review (**Figure 1**) (**Supplementary Table 3**) [1–84].

Because intervention duration and follow-up varied across studies, we applied prespecified extraction rules to avoid unit-of-analysis errors from using multiple time points from the same study. For efficacy outcomes reported at several time points, we extracted data for the prespecified primary endpoint or, when applicable, the end-of-treatment assessment. If no primary endpoint was specified, we used the follow-up closest to 12 weeks. If 12-week data were unavailable, we used the follow-up closest to completion of the intervention course. For complete healing, ulcer area reduction, and time to healing, only one time point per outcome from each study was included in the main analysis.

For safety and prognostic outcomes, including adverse events and amputation, cumulative event counts were extracted preferentially. When several time points were reported, we used the time point selected for the primary efficacy outcome whenever possible. If safety outcomes were reported only at final follow-up, cumulative events over the longest available follow-up were extracted. This approach was intended to improve comparability of outcome timing across studies and to preserve the independence of effect estimates by avoiding repeated inclusion of the same trial at multiple time points.

Overall, 84 studies comprising 7,232 participants with diabetic foot ulcers were included. Adjunctive therapies added to standard of care were grouped into eight active intervention categories: OXY, DEV, GFB, PLA, MSS, CAD, TBA, and NND. SOC served as the common comparator in both the network meta-analysis and the conventional pairwise meta-analyses.

The included studies were conducted across diverse countries and regions, most frequently in the United States (27 studies), India (12 studies), China (7 studies), and Egypt (5 studies); the remainder were conducted in other countries or as multinational collaborations. Intervention duration varied by treatment category: 2–12 weeks for oxygen-based therapies, 2–20 weeks for device-based and physical wound therapies, 3–20 weeks for growth factor and blood-derived therapies, 8–16 weeks for biomaterial and matrix products, 9–16 weeks for placental and perinatal tissue-derived products, 6–12 weeks for cell-based and adipose-derived therapies, 6–12 weeks for topical pharmacologic and bioactive agents, and 6–16 weeks for nanotechnology-based, natural product, and bioactive dressing therapies.

Participants were mainly middle-aged or older adults with diabetic foot ulcers. Mean age ranged from approximately 49 to 71 years across studies, and men represented the majority of participants in most trials. Baseline ulcer area varied widely, although most studies reported mean baseline wound areas of approximately 2–8 cm². Detailed characteristics of all included studies are presented in **Supplementary Figure 1**.

### 3.2 Risk-of-bias assessment

Risk of bias was assessed with the RoB 2 tool. Overall, 43 randomized controlled trials were judged to be at low risk of bias, 20 raised some concerns, and 21 were judged to be at high risk. Common sources of bias included incomplete reporting of random sequence generation or allocation concealment, limited feasibility of blinding during intervention delivery, missing outcome data in some trials, and unclear risk of selective reporting.

Because adjunctive treatments for diabetic foot ulcers often involve devices, dressings, or topical procedures, blinding of participants and care providers was difficult in several trials. Accordingly, concerns were frequent in the domains of deviations from intended interventions and outcome measurement. The risk-of-bias assessments informed the subsequent GRADE/CINeMA certainty ratings and were considered when interpreting treatment rankings and indirect comparisons. The RoB 2 summary is shown in **Supplementary Figure 1**.

### 3.3 Pairwise meta-analysis

**Figure 2:**
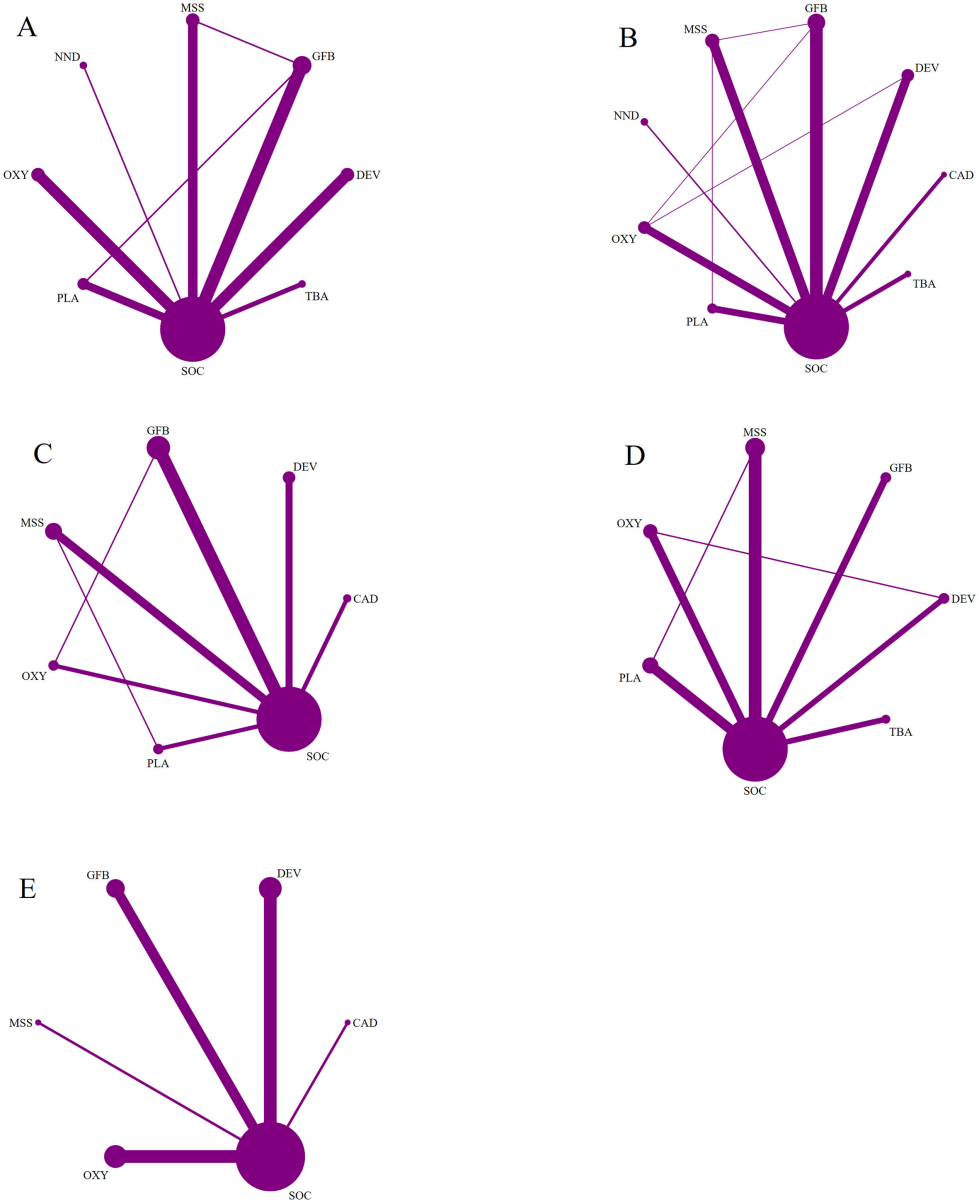
Network map showing connections between various adjuvant therapies for below-ankle diabetic foot ulcers and standard care: **(A)** Reduction in ulcer area; **(B)** Complete wound healing; **(C)** Healing time; **(D)** Adverse Event; **(E)** Amputation rate.

To contextualize the network meta-analysis and strengthen interpretation of the direct evidence, we conducted conventional pairwise meta-analyses comparing each of the eight adjunctive therapy categories with standard of care (SOC). Five outcomes were assessed: ulcer area reduction, complete ulcer healing, time to healing, adverse events, and amputation (**Figure 2**). Continuous outcomes were summarized as standardized mean differences (SMDs) with 95% confidence intervals (CIs), and dichotomous outcomes as odds ratios (ORs) with 95% CIs. Model selection was guided by between-study heterogeneity, with random-effects models used preferentially when I² exceeded 50%. Pairwise meta-analysis results are shown in forest plots, and summary estimates are reported in **Supplementary Table 4**.

#### 3.3.1 Ulcer area reduction

For ulcer area reduction, seven intervention categories had direct pairwise comparisons with SOC. OXY, DEV, GFB, PLA, and MSS were each associated with significantly greater reductions in ulcer area than SOC. The largest pooled effect was observed for DEV (7 studies; SMD = 1.52, 95% CI 0.61 to 2.42; P = 0.001; I²=94.5%), suggesting that device-based and physical adjunctive therapies may substantially reduce wound area. PLA also showed a significant benefit (5 studies; SMD = 1.25, 95% CI 0.03 to 2.47; P = 0.044; I²=94.5%), although heterogeneity was substantial, likely reflecting differences in placenta-derived products, preparation methods, and treatment regimens.

Significant effects were also found for GFB (8 studies; SMD = 0.74, 95% CI 0.19 to 1.29; P = 0.008; I²=89.9%), MSS (6 studies; SMD = 0.45, 95% CI 0.08 to 0.82; P = 0.016; I²=65.9%), and OXY (7 studies; SMD = 0.44, 95% CI 0.11 to 0.78; P = 0.010; I²=67.8%). In contrast, TBA (3 studies; SMD = 0.13, 95% CI −0.26 to 0.52; P = 0.514; I²=17.4%) and NND (1 study; SMD = 0.24, 95% CI −0.55 to 1.03; P = 0.550) did not differ significantly from SOC. The corresponding forest plots are shown in **Supplementary Figures S2–S8**.

#### 3.3.2 Complete ulcer healing

For complete ulcer healing, all eight intervention categories were included in the pairwise meta-analysis. Compared with SOC, all categories except CAD and NND significantly increased the odds of complete healing. OXY was associated with higher odds of complete healing than SOC (11 studies; OR = 2.52, 95% CI 1.57 to 4.04; P<0.001; I²=32.5%), suggesting a potential healing benefit of oxygen-based adjunctive therapies. MSS also showed a consistent benefit (13 studies; OR = 2.08, 95% CI 1.65 to 2.62; P<0.001; I²=0.0%), with negligible between-study heterogeneity.

Significant effects were also observed for DEV (12 studies; OR = 1.79, 95% CI 1.36 to 2.37; P<0.001; I²=38.2%), PLA (9 studies; OR = 1.77, 95% CI 1.32 to 2.38; P<0.001; I²=0.0%), TBA (6 studies; OR = 1.58, 95% CI 1.16 to 2.14; P = 0.003; I²=0.0%), and GFB (17 studies; OR = 1.41, 95% CI 1.17 to 1.71; P<0.001; I²=0.0%). CAD showed a non-significant trend toward improved healing (5 studies; OR = 1.56, 95% CI 0.90 to 2.69; P = 0.111; I²=0.0%). Evidence for NND was sparse and showed no significant effect (2 studies; OR = 1.54, 95% CI 0.75 to 3.18; P = 0.241; I²=0.0%). The corresponding forest plots are shown in **Supplementary Figures S9–S16**.

#### 3.3.3 Time to healing

Six intervention categories had direct comparisons with SOC for time to healing. A negative SMD indicates shorter healing time with the adjunctive intervention. CAD, GFB, and MSS were each associated with significantly shorter healing time than SOC. The largest effect was observed for CAD (3 studies; SMD=−1.55, 95% CI −2.45 to −0.65; P = 0.001; I²=74.5%). Significant reductions were also observed for GFB (9 studies; SMD=−1.17, 95% CI −1.83 to −0.52; P<0.001; I²=90.3%) and MSS (6 studies; SMD=−0.89, 95% CI −1.38 to −0.41; P<0.001; I²=76.9%).

OXY (3 studies; SMD=−0.53, 95% CI −1.09 to 0.03; P = 0.062; I²=67.5%), DEV (5 studies; SMD=−0.77, 95% CI −1.58 to 0.05; P = 0.065; I²=97.1%), and PLA (3 studies; SMD=−0.87, 95% CI −1.77 to 0.02; P = 0.055; I²=82.8%) showed trends toward shorter healing time, but the effects were not statistically significant. The corresponding forest plots are shown in **Supplementary Figures S17–S22**.

#### 3.3.4 Adverse events

Six intervention categories were included in the pairwise meta-analysis of adverse events. PLA was associated with a lower risk of adverse events than SOC (7 studies; OR = 0.51, 95% CI 0.30 to 0.89; P = 0.017; I²=5.4%), with little between-study heterogeneity. No statistically significant differences were observed for OXY (6 studies; OR = 0.86, 95% CI 0.56 to 1.33; P = 0.497; I²=0.0%), DEV (4 studies; OR = 1.16, 95% CI 0.91 to 1.47; P = 0.227; I²=0.0%), GFB (5 studies; OR = 1.07, 95% CI 0.75 to 1.53; P = 0.710; I²=0.0%), MSS (9 studies; OR = 0.77, 95% CI 0.52 to 1.14; P = 0.189; I²=0.0%), or TBA (4 studies; OR = 0.89, 95% CI 0.66 to 1.20; P = 0.448; I²=0.0%). The corresponding forest plots are shown in **Supplementary Figures S23–S28**.

#### 3.3.5 Amputation

Five intervention categories had direct comparisons with SOC for amputation. OXY and GFB were each associated with a significantly lower risk of amputation. Compared with SOC, OXY reduced the odds of amputation (5 studies; OR = 0.27, 95% CI 0.10 to 0.71; P = 0.008; I²=61.4%), suggesting that oxygen-based adjunctive therapies may reduce severe adverse outcomes. GFB showed a similar protective association (4 studies; OR = 0.32, 95% CI 0.13 to 0.77; P = 0.011; I²=0.0%). DEV showed a non-significant trend toward lower amputation risk (5 studies; OR = 0.72, 95% CI 0.46 to 1.14; P = 0.160; I²=30.3%). Evidence for MSS (1 study; OR = 0.83, 95% CI 0.05 to 13.70; P = 0.896) and CAD (1 study; OR = 0.50, 95% CI 0.04 to 6.44; P = 0.595) was sparse and imprecise, precluding firm conclusions. The corresponding forest plots are shown in **Supplementary Figures S29–S33**.

#### 3.3.6 Subgroup analyses

Subgroup analyses were performed by specific treatment modality to examine whether effects varied across interventions within the same broad category. Effect estimates differed for some outcomes, suggesting that treatment technology, product source, treatment frequency, and treatment duration may modify therapeutic effects. Because several subgroups included few studies, between-subgroup comparisons had limited statistical power. These analyses were therefore used mainly to explore sources of heterogeneity and to support interpretation of the main findings, rather than as an independent basis for treatment ranking. The corresponding results are shown in **Supplementary Figure S34–S57**.

#### 3.3.7 Sensitivity analyses

Robustness of the pairwise meta-analyses was examined using leave-one-out sensitivity analyses. Overall, the direction of the pooled estimates remained broadly consistent with the main analyses after sequential removal of individual studies, indicating reasonable stability of the principal findings. For ulcer area reduction, complete ulcer healing, and amputation, effect sizes fluctuated after removal of some studies, but the direction of effect did not materially change. For comparisons with few studies or substantial heterogeneity at baseline, leave-one-out analyses indicated greater susceptibility to the influence of individual studies. These findings should therefore be interpreted alongside the number of studies, sample size, study quality, and clinical heterogeneity. The corresponding analyses are shown in **Supplementary Figures S58–S85**.

#### 3.3.8 Meta-regression

Because several outcomes and intervention categories included few studies, meta-regression was conducted as an exploratory analysis. Follow-up duration was entered as a study-level covariate to examine whether it accounted for between-study heterogeneity in the pairwise meta-analyses. Results are presented in **Supplementary Table 5**, including the covariate, regression coefficient, standard error, 95% CI, P value, overall model test, residual I², and R² for each analyzable comparison. Overall, follow-up duration showed no stable or consistent association with pooled effect estimates, suggesting that it did not explain a substantial proportion of the observed heterogeneity in the available data. Because these analyses used aggregate study-level data, however, they cannot substitute for individual participant data analyses and remain vulnerable to ecological bias, particularly when few studies are available. The corresponding plots are shown in **Supplementary Figures S86–S109**.

#### 3.3.9 Publication bias and small-study effects

For comparisons with more than 10 studies, funnel plots were generated to assess publication bias and small-study effects. Most plots showed no clear asymmetry, although interpretation was limited where study numbers were small. Publication bias assessments were therefore treated as supportive evidence and interpreted alongside study size, the distribution of effect estimates, and certainty of evidence. The corresponding plots are shown in **Supplementary Figures S110–S113**.

### 3.4 Consistency and heterogeneity

Closed loops were present in the networks for ulcer area reduction, complete healing, and time to healing; global inconsistency tests were therefore performed for these outcomes. These tests showed no statistically significant inconsistency at the 0.05 level (**Supplementary Table 6**), although some node-splitting and loop-specific analyses suggested borderline inconsistency (**Supplementary Tables S7–S12**). Accordingly, the consistency model was used for the primary analyses, with cautious interpretation and downgrading for comparisons supported by sparse closed loops, limited direct evidence, or borderline inconsistency signals.

For adverse events and amputation, the networks contained too few closed loops to permit reliable global, local, or loop-specific inconsistency testing. Network estimates for these outcomes therefore relied mainly on the transitivity assumption and the clinical comparability of direct and indirect evidence, and should not be interpreted as having been formally validated by consistency testing. Based on the baseline characteristics summarized in **Supplementary Table 3**, studies were broadly comparable in age and follow-up duration. In the GRADE/CINeMA assessment, relevant comparisons were downgraded for indirectness and imprecision where appropriate, and rankings for safety and amputation outcomes were interpreted as exploratory.

### 3.5 Network meta-analysis

Random-effects network meta-analyses were performed for ulcer area reduction, complete ulcer healing, time to healing, adverse events, and amputation across the eight intervention categories.

#### 3.5.1 Ulcer area reduction

**Figure 3:**
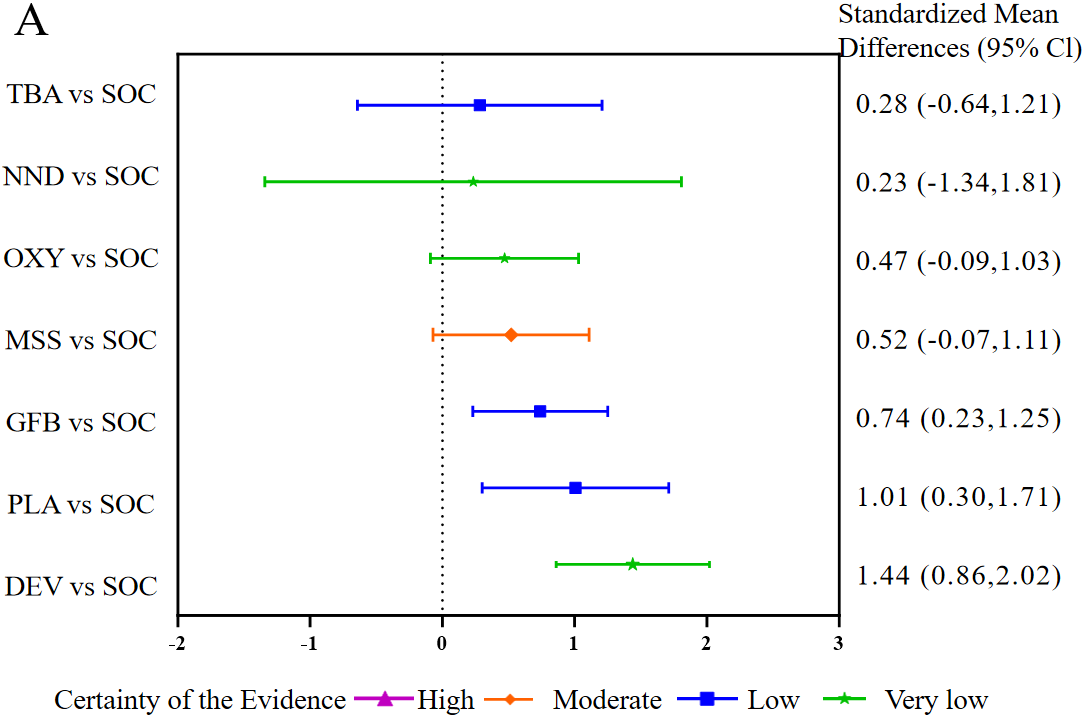
Forest plot of standardized mean differences (95% CI) for ulcer area reduction across interventions versus standard care. Interventions are listed in descending order of SUCRA values (**Supplementary Figure 114**). Effect estimates are from contingency tables (**Supplementary Table 13**), with evidence certainty detailed in (**Supplementary Table 23**). **(A)** corresponds to ulcer area reduction.

For ulcer area reduction, 35 studies contributed data on seven intervention categories, comprising 2,724 adults with diabetes and foot ulcers below the ankle. Compared with SOC, DEV was associated with a greater reduction in ulcer area (SMD = 1.44, 95% CI 0.86 to 2.02), although the certainty of evidence was very low. PLA (SMD = 1.01, 95% CI 0.30 to 1.71) and GFB (SMD = 0.74, 95% CI 0.23 to 1.25) also showed potential benefit, supported by low-certainty evidence (**Figure 3**).

Based on SUCRA values, device-based and physical wound therapies (DEV) ranked highest (95.3%), followed by placental and perinatal tissue-derived products (PLA; 76.4%) and growth factor and blood-derived therapies (GFB; 61.6%). However, given the low overall certainty of the evidence, these rankings should be interpreted as exploratory and should not be used alone to guide clinical decision-making (**Supplementary Table 13**).

#### 3.5.2 Complete ulcer healing

**Figure 4:**
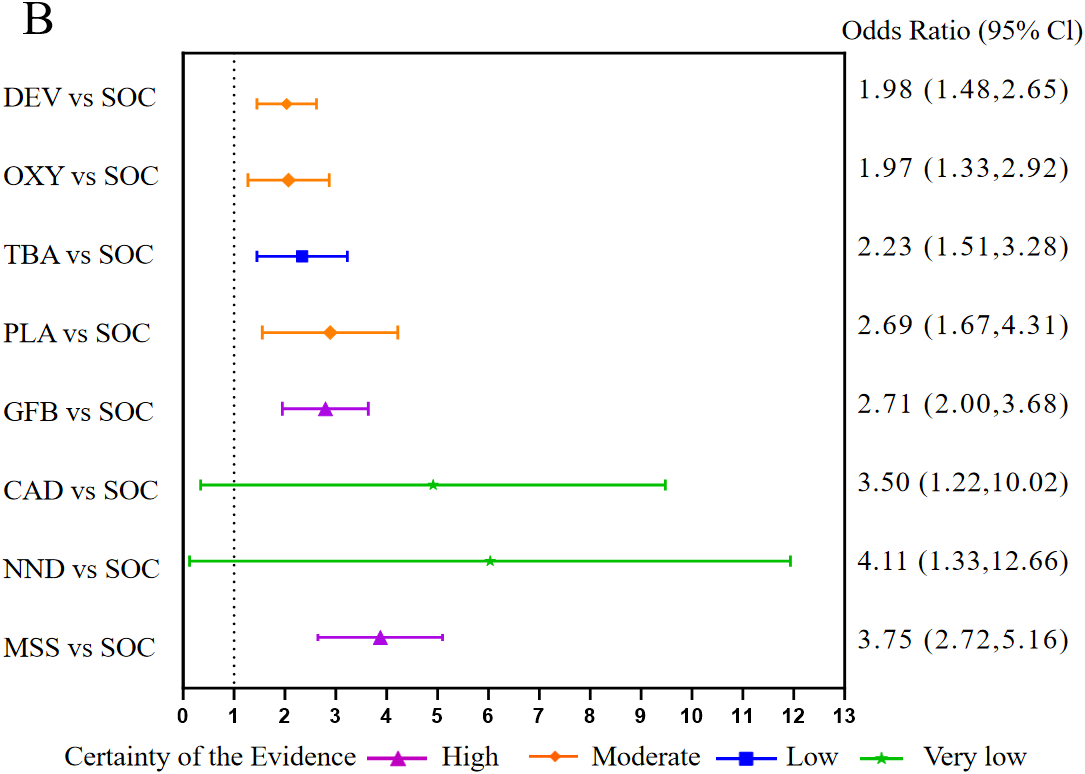
Forest plot of odds ratios (95% CI) for complete wound healing across interventions versus standard care. Interventions are listed in descending order of SUCRA values (**Supplementary Figure 115**). Effect estimates are from contingency tables (**Supplementary Table 14**), with evidence certainty detailed in (**Supplementary Table 24**). **(B)** complete ulcer healing.

For complete ulcer healing, 73 studies contributed data on eight intervention categories, comprising 6,402 adults with diabetes and foot ulcers below the ankle. Compared with SOC, MSS was associated with higher odds of complete healing (OR = 3.75, 95% CI 2.72 to 5.16), supported by high-certainty evidence. NND (OR = 4.11, 95% CI 1.33 to 12.66) and CAD (OR = 3.50, 95% CI 1.22 to 10.02) were also associated with higher odds of complete healing, although the certainty of evidence was very low (**Figure 4**).

Based on SUCRA values, PLA ranked highest (77.9%), followed by MSS (72.1%) and CAD (69.7%). Treatment rankings should be interpreted only as exploratory and considered alongside direct-comparison results, effect estimates with confidence intervals, and certainty of evidence; they should not be used alone as the basis for clinical recommendations (**Supplementary Table 14**).

#### 3.5.3 Time to healing

**Figure 5:**
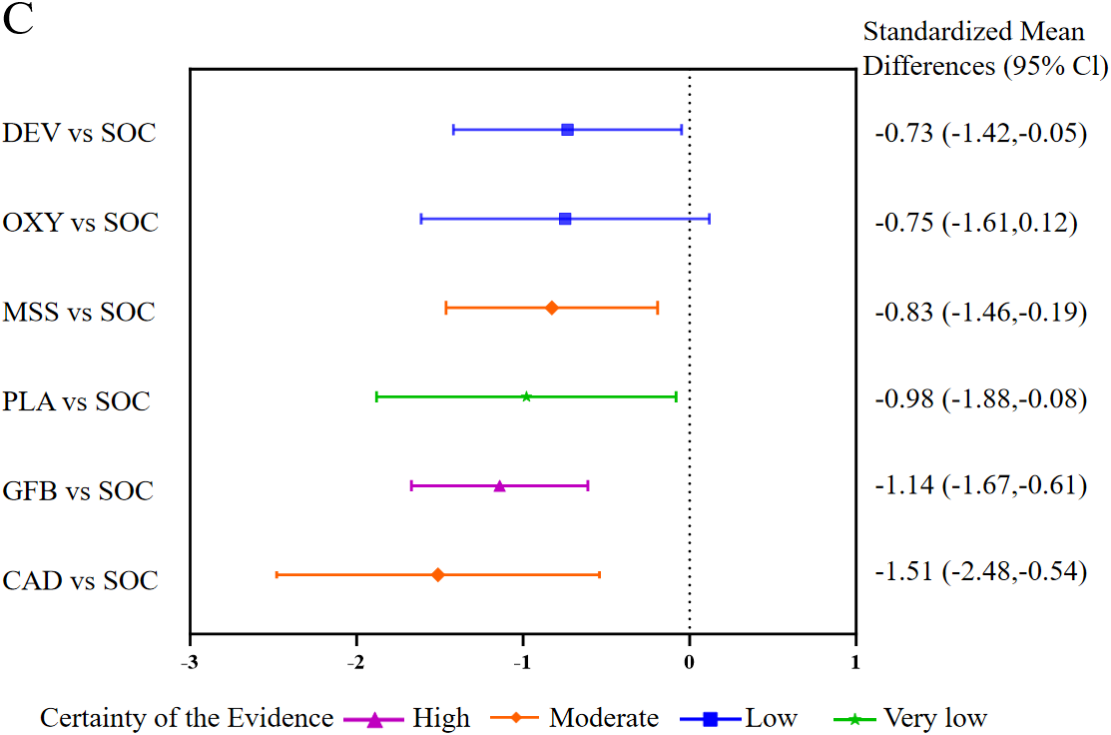
Forest plot of standardized mean differences (95% CI) for healing time across interventions versus standard care.Interventions are listed in descending order of SUCRA values (**Supplementary Figure 116**). Effect estimates are from contingency tables (**Supplementary Table 15**), with evidence certainty detailed in (**Supplementary Table 25**). **(C)** time to healing.

For time to complete healing, 28 studies were included, evaluating six intervention categories in 2,265 adults with diabetes and foot ulcers below the ankle. Negative effect estimates indicate shorter healing time with the adjunctive intervention than with SOC. The network meta-analysis suggested that CAD may shorten time to healing (SMD=−1.51, 95% CI −2.48 to −0.54), supported by moderate-certainty evidence. GFB also showed a potential advantage (SMD=−1.14, 95% CI −1.67 to −0.61), supported by high-certainty evidence, and PLA showed a possible benefit (SMD=−0.98, 95% CI −1.88 to −0.08), although the certainty of evidence was very low (**Figure 5**).

Based on SUCRA values, CAD ranked highest (86.0%), followed by GFB (70.4%) and PLA (59.4%). Because time to healing is highly sensitive to follow-up duration, healing definitions, and assessment frequency, and because the overall certainty of evidence was limited, these rankings should be interpreted as exploratory (**Supplementary Table 15**).

#### 3.5.4 Adverse events

**Figure 6:**
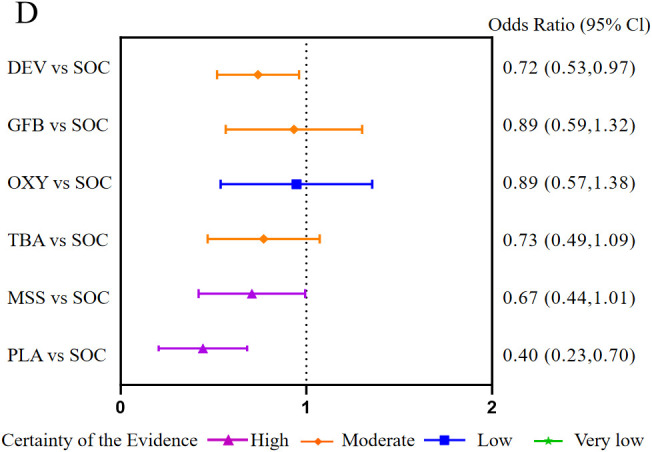
Forest plot of odds ratios (95% CI) for adverse event across interventions versus standard care. Interventions are listed in descending order of SUCRA values (**Supplementary Figure 117**). Effect estimates are from contingency tables (**Supplementary Table 16**), with evidence certainty detailed in (**Supplementary Table 26**). **(D)** adverse events.

For adverse events, 35 studies contributed data on six intervention categories, comprising 3,695 adults with diabetes and foot ulcers below the ankle. In the network meta-analysis, PLA was associated with a lower risk of adverse events (OR = 0.40, 95% CI 0.23 to 0.70), supported by high-certainty evidence. MSS (OR = 0.67, 95% CI 0.44 to 1.01; high-certainty evidence) and TBA (OR = 0.73, 95% CI 0.49 to 1.09; moderate-certainty evidence) also suggested possible reductions in adverse events. Given the variation across studies in adverse-event definitions, severity grading, and active surveillance methods, this outcome should be interpreted cautiously (**Figure 6**).

Based on SUCRA values, PLA ranked highest (98.7%), followed by MSS (73.7%) and TBA (66.8%). However, because the safety networks contained too few closed loops and adverse-event definitions and reporting completeness varied across studies, these rankings should be considered descriptive only and should not be interpreted as evidence of definitive safety superiority for any treatment category (**Supplementary Table 16**).

#### 3.5.5 Amputation

**Figure 7:**
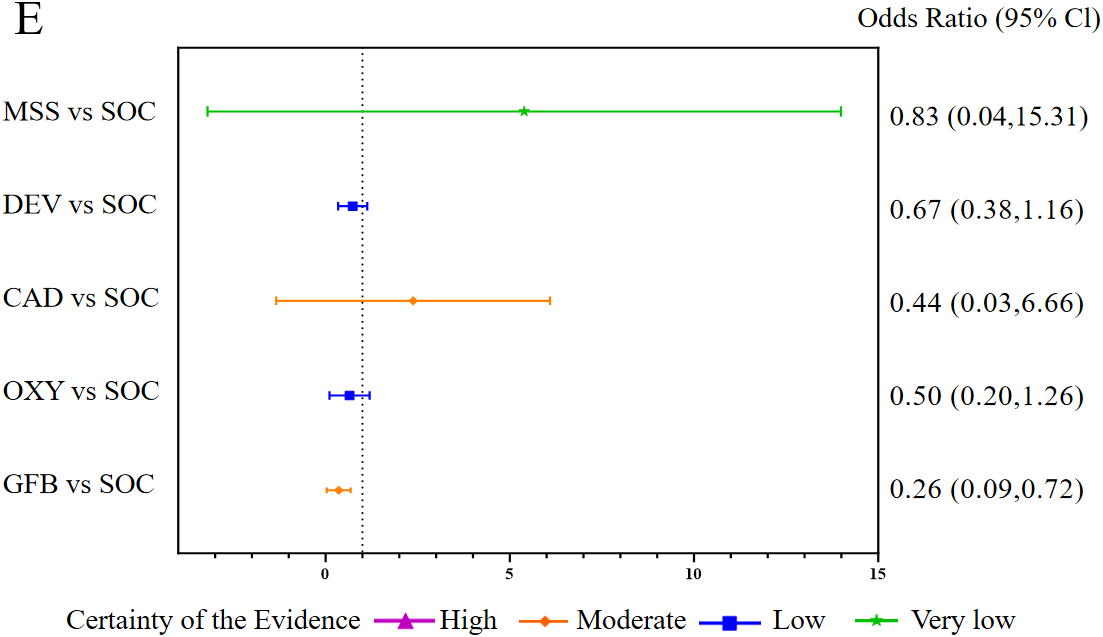
Forest plot of odds ratios (95% CI) for amputation Rate across interventions versus standard care. Interventions are listed in descending order of SUCRA values (**Supplementary Figure 118**). Effect estimates are from contingency tables (**Supplementary Table 17**), with evidence certainty detailed in (**Supplementary Table 27**). **(E)** amputation rate.

For amputation, 16 studies contributed data on five intervention categories, comprising 2,022 adults with diabetes and foot ulcers below the ankle. Compared with SOC, OXY may reduce amputation risk (OR = 0.50, 95% CI 0.20 to 1.26), supported by low-certainty evidence. GFB also showed a potential protective effect (OR = 0.26, 95% CI 0.09 to 0.72), supported by moderate-certainty evidence. Because amputation events were few and the network contained too few closed loops for reliable inconsistency assessment, these findings should be interpreted as exploratory and of limited certainty (**Figure 7**).

Based on SUCRA values, OXY ranked highest (84.8%), followed by GFB (72.4%). Because amputation events were few and confidence intervals were wide for some comparisons, the evidence remains insufficient for firm conclusions; these rankings should be considered exploratory (**Supplementary Table 17**).

### 3.6 Meta-regression and publication bias

Key baseline characteristics showed no clear systematic imbalance across treatment groups. However, reporting of ulcer severity, infection or ischaemia status, adherence to offloading, and SOC components was often incomplete, leaving some uncertainty around the transitivity assumption. Network meta-regression showed no consistent association between follow-up duration and most outcomes (**Supplementary Tables S18–S22**). Because these analyses were based on study-level data and had limited statistical power, the influence of unmeasured effect modifiers cannot be excluded.

Comparison-adjusted funnel plots were generated for outcomes with a sufficient number of studies to assess publication bias and small-study effects. Overall, the plots showed no clear asymmetry or extreme outliers. However, because some comparisons included few studies and the network geometry was uneven, these assessments had limited power and cannot rule out publication bias or small-study effects. The comparison-adjusted funnel plots are shown in **Supplementary Figures S119–S123**.

### 3.7 Certainty of evidence: GRADE/CINeMA assessment

The certainty of evidence for the network meta-analysis was assessed using the GRADE framework and CINeMA. Across the five outcome networks, 18 treatment comparisons were rated as high certainty, 31 as moderate certainty, 39 as low certainty, and 33 as very low certainty. Higher-certainty comparisons were generally supported by direct head-to-head evidence and confidence intervals that did not cross either the line of no effect or the prespecified threshold for a clinically important effect. Low- and very-low-certainty ratings were most often attributable to within-study bias, sparse networks, a high proportion of indirect evidence, imprecision, borderline inconsistency, or possible small-study effects.

Overall, several intervention categories showed potential benefits across multiple outcomes. However, because certainty was limited for many comparisons, SUCRA rankings and network estimates should be interpreted as supportive and exploratory rather than definitive. Outcome-specific certainty ratings are provided in **Supplementary Tables S23–S27**.

## 4 Discussion

### 4.1 Principal findings

In this updated network meta-analysis, adjunctive treatments for diabetic foot ulcers were reclassified into clinically interpretable categories, and conventional pairwise meta-analyses were added to appraise the direct evidence for each intervention category versus standard of care. Overall, several adjunctive therapies provided additional benefit over standard care for healing-related outcomes, although the relative advantages of individual categories differed by outcome.

In the pairwise meta-analyses, DEV, PLA, GFB, MSS, and OXY significantly improved ulcer area reduction; OXY, MSS, DEV, PLA, TBA, and GFB significantly increased complete ulcer healing; CAD, GFB, and MSS shortened time to healing; and OXY and GFB reduced amputation risk. For adverse events, only PLA was associated with a lower risk than SOC. These findings indicate that the effects of adjunctive therapies for diabetic foot ulcers are outcome dependent, and that no single endpoint adequately captures the clinical value of a treatment category.

The network and pairwise meta-analyses showed broadly similar directions of effect for several outcomes, but differences emerged for complete healing and treatment rankings. In the pairwise analyses, for example, OXY, MSS, DEV, PLA, TBA, and GFB improved complete healing, whereas PLA, MSS, and CAD ranked highest in the network analysis. CAD did not reach statistical significance in the pairwise analysis but ranked highly in the network. These discrepancies suggest that network rankings may be shaped by indirect evidence, network geometry, and intervention grouping. We therefore treated the pairwise meta-analyses as complementary direct evidence and used the network meta-analysis to explore relative rankings, without interpreting SUCRA values as stand-alone evidence of treatment efficacy.

### 4.2 Clinical interpretation across outcomes

For ulcer area reduction, DEV, PLA, GFB, MSS, and OXY each showed a statistically significant advantage over SOC, with the largest effect estimates observed for DEV and PLA. DEV interventions may improve the wound microenvironment through mechanical, physical, or energy-based mechanisms, including stimulation of granulation tissue formation, enhancement of local perfusion, reduction of exudate, and modulation of inflammation (5, 15). PLA products may support chronic wound repair by supplying extracellular matrix components, growth factors, and immunomodulatory mediators, thereby creating a local environment more closely aligned with physiological tissue repair (5, 16). These mechanisms provide biological plausibility for the relatively large short-term effects of DEV and PLA on wound area reduction.

For complete ulcer healing, OXY, MSS, DEV, PLA, TBA, and GFB were each associated with significant benefit. Complete healing is one of the most clinically meaningful endpoints in diabetic foot ulcer trials, as it reflects the final outcome of wound repair more directly than area reduction alone. OXY therapies may act by improving local oxygen availability, enhancing fibroblast function, promoting collagen synthesis, and strengthening host defence against infection. MSS interventions may support wound closure by providing a structural scaffold, facilitating cell migration, and promoting tissue reconstruction (5, 9). GFB therapies may increase repair activity in chronic wounds by supplementing or activating biological signalling pathways involved in wound healing (5).

For time to healing, CAD, GFB, and MSS were associated with significantly shorter healing times. This outcome reflects not only treatment efficiency but also dressing burden, duration of exposure to infection risk, healthcare costs, and quality of life. Notably, some interventions significantly increased complete healing but showed only a trend, or no statistically significant effect, for time to healing, suggesting that the probability and speed of healing may be influenced by different factors. Future trials should report the time from randomization to complete epithelialization more consistently and should standardize follow-up schedules and healing criteria.

Regarding safety, most adjunctive therapies were not associated with a significant increase in adverse events. PLA was associated with a lower risk of adverse events in both the pairwise and network meta-analyses, but this finding should be interpreted cautiously. Adverse-event definitions, severity grading, and active surveillance methods varied across studies; some trials may have focused more on wound healing than on systemic safety outcomes, and the safety network contained too few closed loops for robust inconsistency assessment. Current evidence is therefore insufficient to conclude that any adjunctive therapy category has a definitive safety advantage. Rather, the available data suggest that most therapies did not confer a clear excess risk in randomized trial settings.

For amputation, OXY and GFB showed potential risk reductions. Amputation is among the most severe outcomes of diabetic foot ulcers and is influenced by infection control, vascular status, ulcer depth, osteomyelitis, revascularization, and the quality of multidisciplinary care (3). The effect of adjunctive therapies on amputation is therefore difficult to attribute to a single mechanism. OXY may reduce wound deterioration by improving tissue oxygenation and host defence against infection, whereas GFB may reduce prolonged wound exposure and infection progression by promoting repair. Because amputation events were relatively few, these findings require confirmation in larger trials.

### 4.3 Consistency of findings and clinical implications

The main directions of effect were broadly consistent between the pairwise and network meta-analyses, although not all outcomes and treatment rankings aligned. Pairwise meta-analysis relies on direct evidence and therefore provides a more immediate estimate of each intervention category versus SOC. Network meta-analysis combines direct and indirect evidence and enables comparisons across multiple adjunctive therapies. When the two approaches diverge, interpretation should be guided by the amount of direct evidence, confidence intervals, network geometry, and certainty of evidence, rather than by P values or SUCRA rankings alone.

The relative advantages of the intervention categories differed across outcomes. DEV, PLA, GFB, MSS, and OXY performed well for ulcer area reduction, suggesting that these therapies may improve the local wound repair environment. OXY, MSS, DEV, PLA, TBA, and GFB increased complete healing, indicating that several adjunctive approaches may promote final wound closure through different mechanisms. CAD, GFB, and MSS shortened time to healing, suggesting potential value for patients in whom faster wound repair is a priority. OXY and GFB showed possible benefit in reducing amputation risk, although this finding should be interpreted cautiously because amputation events were limited.

These findings indicate that adjunctive therapies for diabetic foot ulcers should not be judged by a single endpoint. Ulcer area reduction reflects early wound improvement, complete healing captures the final repair outcome, time to healing reflects treatment efficiency and care burden, adverse events inform safety, and amputation represents the most severe clinical prognosis. The clinical value of each adjunctive therapy should therefore be assessed in relation to patient characteristics and treatment goals, rather than selected solely on the basis of a single outcome or SUCRA ranking.

### 4.4 Value of combining network and pairwise meta-analyses

A key methodological feature of this study is the combined use of network meta-analysis and conventional pairwise meta-analysis. Network meta-analysis integrates direct and indirect evidence and permits ranking of multiple interventions, making it useful for assessing which adjunctive therapy may be preferable among several options. In diabetic foot ulcer research, however, limited study numbers, heterogeneous comparators, and incomplete outcome reporting can produce sparse networks with few closed loops. In this setting, treatment rankings based on network meta-analysis alone may be overinterpreted.

Pairwise meta-analysis, by contrast, relies primarily on direct evidence and is better suited to determining whether a given adjunctive therapy category is effective compared with SOC. Its estimates are more immediate and often easier for clinicians to interpret. In this study, pairwise meta-analysis was used to examine the direct effect of each intervention category versus SOC, while network meta-analysis provided an overall view of relative performance across multiple interventions.

This combined approach is particularly useful for adjunctive treatments in diabetic foot ulcers, where interventions are heterogeneous, individual trials are often small, and treatment protocols vary substantially. A single meta-analytic framework may not adequately capture the full evidence base. Used together, pairwise and network meta-analyses provide complementary perspectives and help reduce the uncertainty inherent in any single model.

### 4.5 Sources of heterogeneity and stability of findings

Substantial heterogeneity was observed for some outcomes, particularly ulcer area reduction and time to healing. Several factors may have contributed. First, DFU severity varied across studies, including ulcer area, ulcer duration, infection status, degree of ischaemia, and severity of neuropathy. Second, SOC differed in its components, including debridement, offloading, dressing selection, infection control, vascular assessment, and glycaemic management. Third, intervention protocols varied in treatment frequency, duration, dose, product source, and procedural details. Fourth, outcome assessment was not uniform, with differences in ulcer area measurement, criteria for complete healing, and follow-up timing.

Sensitivity analyses showed that the direction of pooled effects remained broadly consistent after sequential removal of individual studies in most comparisons. However, statistical significance changed after exclusion of some studies in several borderline or highly heterogeneous comparisons. For example, P values crossed 0.05 in some analyses of ulcer area reduction, time to healing, and adverse events, indicating sensitivity to individual studies. For these findings, interpretation should focus on the direction of effect, confidence intervals, and certainty of evidence rather than P values alone.

Meta-regression using follow-up duration as a study-level covariate found no stable association with effect size, suggesting that follow-up duration was unlikely to be the main source of heterogeneity in the available data. Because the analyses were based on aggregate study-level data and several comparisons included few studies, however, statistical power was limited and the influence of other potential effect modifiers cannot be excluded. Future studies should report ulcer severity, infection status, vascular status, adherence to offloading, and SOC components more systematically to allow more reliable exploration of heterogeneity.

### 4.6 Implications for clinical practice

This study suggests that adjunctive therapies, when appropriately added to standard care, may further improve wound healing outcomes in patients with diabetic foot ulcers. Treatment effects, however, were not consistent across outcomes, so clinical selection should be individualized according to the primary therapeutic goal.

If the goal is ulcer area reduction, DEV, PLA, GFB, MSS, and OXY may be relevant options. If the goal is complete healing, OXY, MSS, DEV, PLA, TBA, and GFB showed potential benefit in direct comparisons. If faster healing is the priority, CAD, GFB, and MSS warrant further consideration. For severe outcomes such as amputation, OXY and GFB showed possible protective effects. These choices should be guided by the patient’s ischaemia and infection status, ulcer severity, feasibility of SOC delivery, treatment cost, and certainty of evidence, rather than by SUCRA rankings or any single outcome alone.

No adjunctive therapy should replace core diabetic foot ulcer care. Debridement, offloading, infection control, glycaemic management, vascular assessment, and multidisciplinary care remain the foundation of treatment. Adjunctive therapies should be viewed as additions to SOC, not as stand-alone treatments. In patients with substantial ischaemia, infection, or risk of osteomyelitis, the underlying pathology should be addressed before adjunctive therapy is selected (1).

### 4.7 Relationship to previous studies and guidelines

Previous meta-analyses of adjunctive therapies for diabetic foot ulcers have generally focused on single interventions, such as negative-pressure wound therapy, hyperbaric oxygen therapy, platelet-rich plasma, growth factors, or placenta-derived products. These reviews are useful for assessing whether a specific therapy is effective, but they provide limited insight into the relative value of different adjunctive options. By reclassifying interventions and applying network meta-analysis, the present study integrates a broader range of adjunctive therapies. The addition of conventional pairwise meta-analysis further anchors the findings in direct evidence. Together, these analyses provide a more complete map of the available evidence.

Current guidelines emphasize the central role of SOC and generally make cautious recommendations for selected adjunctive therapies, largely because evidence quality is variable, heterogeneity is substantial, and cost and availability differ across settings. Our findings are broadly consistent with this cautious position. Although several adjunctive therapies showed potential benefit, many comparisons were limited by low certainty of evidence, small sample sizes, or considerable heterogeneity. Accordingly, these findings are best viewed as evidence to inform clinical decision-making, rather than as support for routine use of any adjunctive therapy category in all patients with diabetic foot ulcers.

### 4.8 Strengths and limitations

This study has several strengths. First, interventions were reclassified according to therapeutic mechanism and clinical characteristics, improving the interpretability of the network structure. Second, the combined use of network and pairwise meta-analyses allowed indirect comparisons, relative rankings, and direct evidence to be interpreted together. Third, we assessed several clinically relevant outcomes, including ulcer area reduction, complete healing, time to healing, adverse events, and amputation, providing a broad evaluation of efficacy and safety. Fourth, subgroup analyses, sensitivity analyses, and meta-regression were conducted to examine robustness and explore potential sources of heterogeneity. Fifth, the use of RoB 2 and the GRADE/CINeMA framework supported a cautious and transparent interpretation of the evidence.

Several limitations should also be acknowledged. First, some intervention categories included clinically distinct therapies, which may have introduced within-category heterogeneity. Second, some outcomes and intervention categories were supported by few studies, resulting in imprecise effect estimates. Third, reporting of SOC components and implementation quality was often incomplete, which may have affected comparability across studies. Fourth, follow-up duration, outcome definitions, and measurement methods varied across studies, particularly for time to healing and adverse events. Fifth, some networks lacked closed loops, limiting formal assessment of inconsistency. Finally, although meta-regression and subgroup analyses were used to explore heterogeneity, these analyses were based on limited study-level data and should be considered exploratory.

### 4.9 Future research directions

Future studies of adjunctive therapies for diabetic foot ulcers should place greater emphasis on rigorous trial design and standardized outcome reporting. First, SOC should be clearly defined, with detailed reporting of debridement, offloading, infection control, vascular assessment, glycaemic management, and other core elements of care. Second, key outcome definitions should be standardized, particularly for complete healing, time to healing, adverse events, and amputation. Third, larger trials with longer follow-up are needed to assess long-term outcomes more reliably, including amputation, recurrence, and serious adverse events. Fourth, trial designs should incorporate prespecified patient stratification by ischaemia status, infection severity, ulcer area, and Wagner or University of Texas grade. Finally, future studies should evaluate cost-effectiveness, quality of life, and treatment accessibility to improve the relevance of the evidence for real-world clinical decision-making.

### 4.10 Conclusions

In summary, some adjunctive therapies may improve healing-related outcomes in patients with diabetic foot ulcers when added to standard care. Pairwise and network meta-analyses provided complementary evidence across treatment categories, suggesting potential advantages of OXY, DEV, PLA, GFB, and MSS for different outcomes. These findings should be interpreted cautiously, given study heterogeneity, sparse networks, insufficient closed loops in some analyses, variable certainty of evidence, and small sample sizes for several comparisons. The current evidence is therefore insufficient to support strong recommendations or a definitive treatment hierarchy. Further high-quality, standardized randomized controlled trials with adequate follow-up are needed to define the optimal patient populations, treatment timing, and clinical value of different adjunctive therapies for diabetic foot ulcers.
