# Supplementary material for: Adjunctive therapies added to standard of care for diabetic foot ulcers: a systematic review and network meta-analysis with pairwise comparisons
Source: Front Endocrinol (Lausanne). 2026 Jul 31;17:1891173. doi: 10.3389/fendo.2026.1891173 (PMC13472823; doi:10.3389/fendo.2026.1891173)
Supplement: Supplementary file 1 [file SupplementaryFile1.docx]

Table S1	Adjunctive Therapies Added to Standard of Care for Diabetic Foot Ulcers: A Network Meta-Analysis and Certainty-of-Evidence Assessment

| Section/Topic | Item # | Checklist Item | Reported on Page # |
| --- | --- | --- | --- |
| TITLE |  |  |  |
| Title | 1 | Identify the report as a systematic review *incorporating*  anetwork meta-analysis (or related form of meta-analysis). | 1 |
|  |  |  |  |
| ABSTRACT |  |  |  |
| Structured summary | 2 | Provide a structured summary including, as applicable:  **Background:** main objectives  **Methods:** data sources; study eligibility criteria, participants, and interventions; study appraisal; and *synthesis methods, such as network meta-analysis.*  **Results:** number of studies and participants identified; summary estimates with corresponding confidence/credible intervals; treatment rankings may also be discussed. Authors may choose to summarize pairwise comparisons against a chosen treatment included in their analyses for brevity.  **Discussion/Conclusions:** limitations; conclusions and implications of findings.  **Other:** systematic review registration number with registry name. | 1 |
|  |  |  |  |
| INTRODUCTION |  |  |  |
| Rationale | 3 | Describe the rationale for the review in the context of what is already known*, including mention of why a network meta-analysis has been conducted.* | 1-2 |
| Objectives | 4 | Provide an explicit statement of questions being addressed, with reference to participants, interventions, comparisons, outcomes, and study design (PICOS). | 3 |
|  |  |  |  |
| METHODS |  |  |  |
| Protocol and registration | 5 | Indicate whether a review protocol exists and if and where it can be accessed (e.g., Web address); and, if available, provide registration information, including registration number. | 3 |
| Eligibility criteria | 6 | Specify study characteristics (e.g., PICOS, length of follow-up) and report characteristics (e.g., years considered, language, publication status) used as criteria for eligibility, giving rationale. *Clearly describe eligible treatments included in the treatment network, and note whether any have been clustered or merged into the same node (with justification).* | 3 |
| Information sources | 7 | Describe all information sources (e.g., databases with dates of coverage, contact with study authors to identify additional studies) in the search and date last searched. | 3 |
| Search | 8 | Present full electronic search strategy for at least one database, including any limits used, such that it could be repeated. | 3，Table 2 |
| Study selection | 9 | State the process for selecting studies (i.e., screening, eligibility, included in systematic review, and, if applicable, included in the meta-analysis). | 3-4 |
| Data collection process | 10 | Describe method of data extraction from reports (e.g., piloted forms, independently, in duplicate) and any processes for obtaining and confirming data from investigators. | 4 |
| Data items | 11 | List and define all variables for which data were sought (e.g., PICOS, funding sources) and any assumptions and simplifications made. | 3-4 |
| Geometry of the network | S1 | Describe methods used to explore the geometry of the treatment network under study and potential biases related to it. This should include how the evidence base has been graphically summarized for presentation, and what characteristics were compiled and used to describe the evidence base to readers. | 4 |
| Risk of bias within individual studies | 12 | Describe methods used for assessing risk of bias of individual studies (including specification of whether this was done at the study or outcome level), and how this information is to be used in any data synthesis. | 4 |
| Summary measures | 13 | State the principal summary measures (e.g., risk ratio, difference in means). Also describe the use of additional summary measures assessed, such as treatment rankings and surface under the cumulative ranking curve (SUCRA) values, as well as modified approaches used to present summary findings from meta-analyses. | 4-5 |
| Planned methods of analysis | 14 | Describe the methods of handling data and combining results of studies for each network meta-analysis. This should include, but not be limited to:   - Handling of multi-arm trials; - Selection of variance structure; - Selection of prior distributions in Bayesian analyses; and - Assessment of model fit. | 4-5 |
| Assessment of Inconsistency | S2 | Describe the statistical methods used to evaluate the agreement of direct and indirect evidence in the treatment network(s) studied. Describe efforts taken to address its presence when found. | 4 |
| Risk of bias across studies | 15 | Specify any assessment of risk of bias that may affect the cumulative evidence (e.g., publication bias, selective reporting within studies). | 4-5 |
| Additional analyses | 16 | Describe methods of additional analyses if done, indicating which were pre-specified. This may include, but not be limited to, the following:   - Sensitivity or subgroup analyses; - Meta-regression analyses; - Alternative formulations of the treatment network; and - Use of alternative prior distributions for Bayesian analyses (if applicable). | 4-5 |
| RESULTS† |  |  |  |
| Study selection | 17 | Give numbers of studies screened, assessed for eligibility, and included in the review, with reasons for exclusions at each stage, ideally with a flow diagram. | 6-7.Figure 1 |
| Presentation of network structure | S3 | Provide a network graph of the included studies to enable visualization of the geometry of the treatment network. | 9.Figure 3 |
| Summary of network geometry | S4 | Provide a brief overview of characteristics of the treatment network. This may include commentary on the abundance of trials and randomized patients for the different interventions and pairwise comparisons in the network, gaps of evidence in the treatment network, and potential biases reflected by the network structure. | 8-9 |
| Study characteristics | 18 | For each study, present characteristics for which data were extracted (e.g., study size, PICOS, follow-up period) and provide the citations. | 8,Table 3 |
| Risk of bias within studies | 19 | Present data on risk of bias of each study and, if available, any outcome level assessment. | 7-8,Figure 2 |
| Results of individual studies | 20 | For all outcomes considered (benefits or harms), present, for each study: 1) simple summary data for each intervention group, and 2) effect estimates and confidence intervals. *Modified approaches may be needed to deal with information from larger networks.* | 10-14 |
| Synthesis of results | 21 | Present results of each meta-analysis done, including confidence/credible intervals. In larger networks, authors may focus on comparisons versus a particular comparator (e.g. placebo or standard care), with full findings presented in an appendix. League tables and forest plots may be considered to summarize pairwise comparisons. If additional summary measures were explored (such as treatment rankings), these should also be presented. | 10-14,  Table4-8 |
| Exploration for inconsistency | S5 | Describe results from investigations of inconsistency. This may include such information as measures of model fit to compare consistency and inconsistency models, *P* values from statistical tests, or summary of inconsistency estimates from different parts of the treatment network. | 14 |
| Risk of bias across studies | 22 | Present results of any assessment of risk of bias across studies for the evidence base being studied. | 15 |
| Results of additional analyses | 23 | Give results of additional analyses, if done (e.g., sensitivity or subgroup analyses, meta-regression analyses*, alternative network geometries studied, alternative choice of prior distributions for Bayesian analyses,* and so forth). | 14-15,  Table14-23 |
|  |  |  |  |
| DISCUSSION |  |  |  |
| Summary of evidence | 24 | Summarize the main findings, including the strength of evidence for each main outcome; consider their relevance to key groups (e.g., healthcare providers, users, and policy-makers). | 15-16 |
| Limitations | 25 | Discuss limitations at study and outcome level (e.g., risk of bias), and at review level (e.g., incomplete retrieval of identified research, reporting bias). *Comment on the validity of the assumptions, such as transitivity and consistency. Comment on any concerns regarding network geometry (e.g., avoidance of certain comparisons).* | 18-19 |
| Conclusions | 26 | Provide a general interpretation of the results in the context of other evidence, and implications for future research. | 19 |
|  |  |  |  |
| FUNDING |  |  |  |
| Funding | 27 | Describe sources of funding for the systematic review and other support (e.g., supply of data); role of funders for the systematic review. This should also include information regarding whether funding has been received from manufacturers of treatments in the network and/or whether some of the authors are content experts with professional conflicts of interest that could affect use of treatments in the network. | 19 |

PICOS = population, intervention, comparators, outcomes, study design.

* Text in italics indicate S wording specific to reporting of network meta-analyses that has been added to guidance from the PRISMA statement.

† Authors may wish to plan for use of appendices to present all relevant information in full detail for items in this section.

| **Table S2.Literature Search Strategy** | |
| --- | --- |
| **Pubmed** | ( "Diabetic Foot"[Mesh] OR "Foot Ulcer"[Mesh] OR "diabetic foot ulcer*"[tiab] OR "diabetic foot wound*"[tiab] OR DFU[tiab] OR ( diabet*[tiab] AND foot[tiab] AND (ulcer*[tiab] OR wound*[tiab]) ) ) AND ( "Platelet-Rich Plasma"[Mesh] OR "platelet-rich plasma"[tiab] OR "platelet rich plasma"[tiab] OR PRP[tiab] OR "Growth Substances"[Mesh] OR "growth factor*"[tiab] OR PDGF[tiab] OR becaplermin[tiab] OR EGF[tiab] OR rhEGF[tiab] OR FGF[tiab] OR VEGF[tiab] OR "Skin, Artificial"[Mesh] OR "skin substitute*"[tiab] OR "artificial skin"[tiab] OR "dermal substitute*"[tiab] OR "bioengineered skin"[tiab] OR "tissue-engineered skin"[tiab] OR "acellular dermal matrix"[tiab] OR "extracellular matrix"[tiab] OR collagen[tiab] OR "collagen dressing*"[tiab] OR "collagen matrix"[tiab] OR "amniotic membrane"[tiab] OR "placental membrane"[tiab] OR "placental tissue"[tiab] OR "placental-derived"[tiab] OR "placenta-derived"[tiab] OR amnion[tiab] OR chorion[tiab] OR "umbilical cord"[tiab] OR "Bandages"[Mesh] OR "Occlusive Dressings"[Mesh] OR dressing*[tiab] OR hydrogel*[tiab] OR alginate[tiab] OR "alginate dressing*"[tiab] OR foam[tiab] OR "foam dressing*"[tiab] OR silver[tiab] OR "silver dressing*"[tiab] OR honey[tiab] OR "honey dressing*"[tiab] OR "hyaluronic acid"[tiab] OR carboxymethylcellulose[tiab] OR CMC[tiab] OR hydrofiber[tiab] OR "sucrose octasulfate"[tiab] OR NOSF[tiab] OR "nano-oligosaccharide factor"[tiab] OR "Negative-Pressure Wound Therapy"[Mesh] OR "negative pressure wound therapy"[tiab] OR NPWT[tiab] OR "vacuum-assisted closure"[tiab] OR VAC[tiab] OR "topical negative pressure"[tiab] OR "Hyperbaric Oxygenation"[Mesh] OR "hyperbaric oxygen"[tiab] OR HBOT[tiab] OR "topical oxygen"[tiab] OR "topical oxygen therapy"[tiab] OR "continuous diffusion of oxygen"[tiab] OR CDO[tiab] OR "cyclical topical wound oxygen"[tiab] OR TWO2[tiab] OR "local oxygen"[tiab] OR "oxygen therapy"[tiab] OR "ozone therapy"[tiab] OR ozone[tiab] OR "cold atmospheric plasma"[tiab] OR "cold plasma"[tiab] OR "nitric oxide"[tiab] ) AND ( randomized controlled trial[pt] OR controlled clinical trial[pt] OR randomized[tiab] OR randomised[tiab] OR randomly[tiab] OR trial[tiab] OR placebo[tiab] ) NOT ( animals[mh] NOT humans[mh] ) 368 |
| **Web of Science** | TS=("diabetic foot ulcer*" OR "diabetic foot wound*" OR DFU  OR (diabet* NEAR/3 foot NEAR/3 ulcer*)  OR (diabet* NEAR/3 foot NEAR/3 wound*))  AND  TS=("platelet-rich plasma" OR "platelet rich plasma" OR PRP  OR "platelet-rich fibrin" OR "platelet rich fibrin" OR L-PRF  OR "growth factor*" OR "platelet-derived growth factor" OR PDGF  OR becaplermin  OR "epidermal growth factor" OR EGF OR rhEGF  OR "fibroblast growth factor" OR FGF  OR "vascular endothelial growth factor" OR VEGF  OR "artificial skin" OR "skin substitute*" OR "dermal substitute*"  OR "bioengineered skin" OR "tissue-engineered skin"  OR "acellular dermal matrix" OR "extracellular matrix"  OR collagen OR "collagen dressing*" OR "collagen matrix"  OR "amniotic membrane" OR "placental membrane"  OR "placental-derived" OR "placenta-derived"  OR amnion OR chorion OR "umbilical cord"  OR hydrogel* OR alginate OR "alginate dressing*"  OR foam OR "foam dressing*"  OR silver OR "silver dressing*"  OR honey OR "honey dressing*"  OR "hyaluronic acid"  OR carboxymethylcellulose OR CMC OR hydrofiber  OR "sucrose octasulfate" OR NOSF OR "nano-oligosaccharide factor"  OR "negative pressure wound therapy" OR "negative-pressure wound therapy"  OR NPWT OR "vacuum-assisted closure" OR "vacuum assisted closure" OR VAC  OR "hyperbaric oxygen" OR "hyperbaric oxygen therapy" OR HBOT  OR "topical oxygen" OR "topical oxygen therapy"  OR "continuous diffusion of oxygen" OR CDO  OR "cyclical topical wound oxygen" OR TWO2  OR "ozone therapy" OR ozone  OR "cold atmospheric plasma" OR "cold plasma"  OR "nitric oxide")  AND  TS=(randomized OR randomised OR randomly OR placebo OR trial) |
| **Cochrane** | #1 MeSH descriptor: [Diabetic Foot] explode all trees  #2 MeSH descriptor: [Foot Ulcer] explode all trees  #3 ("diabetic foot" OR "diabetic feet" OR "diabetic foot ulcer" OR "diabetic foot wound" OR DFU OR (diabet* NEAR/3 foot NEAR/3 ulcer) OR (diabet NEAR/3 foot NEAR/3 wound)):ti,ab,kw  #4 #1 OR #2 OR #3  #5 MeSH descriptor: [Platelet-Rich Plasma] explode all trees  #6 ("platelet-rich plasma" OR "platelet rich plasma" OR PRP OR "platelet-rich fibrin" OR "platelet rich fibrin" OR "leukocyte and platelet-rich fibrin" OR "leukocyte- and platelet-rich fibrin" OR L-PRF):ti,ab,kw  #7 MeSH descriptor: [Growth Substances] explode all trees  #8 ("growth factor" OR "platelet-derived growth factor" OR PDGF OR becaplermin OR "epidermal growth factor" OR EGF OR rhEGF OR "fibroblast growth factor" OR FGF OR "vascular endothelial growth factor" OR VEGF):ti,ab,kw  #9 MeSH descriptor: [Skin, Artificial] explode all trees  #10 ("artificial skin" OR "skin substitute" OR "dermal substitute" OR "bioengineered skin" OR "tissue-engineered skin" OR "acellular dermal matrix" OR "dermal matrix" OR "extracellular matrix" OR ECM OR collagen OR "collagen dressing" OR "collagen matrix" OR "amniotic membrane" OR "placental membrane" OR "placental tissue" OR "placental-derived" OR "placenta-derived" OR amnion OR chorion OR "umbilical cord"):ti,ab,kw  #11 MeSH descriptor: [Bandages] explode all trees  #12 MeSH descriptor: [Occlusive Dressings] explode all trees  #13 (dressing* OR hydrogel OR alginate OR "alginate dressing" OR foam OR "foam dressing" OR silver OR "silver dressing" OR honey OR "honey dressing" OR "hyaluronic acid" OR carboxymethylcellulose OR CMC OR hydrofiber OR "sucrose octasulfate" OR NOSF OR "nano-oligosaccharide factor"):ti,ab,kw  #14 MeSH descriptor: [Negative-Pressure Wound Therapy] explode all trees  #15 ("negative pressure wound therapy" OR "negative-pressure wound therapy" OR NPWT OR "vacuum-assisted closure" OR "vacuum assisted closure" OR VAC OR "topical negative pressure"):ti,ab,kw  #16 MeSH descriptor: [Hyperbaric Oxygenation] explode all trees  #17 ("hyperbaric oxygen" OR "hyperbaric oxygen therapy" OR HBOT OR "topical oxygen" OR "topical oxygen therapy" OR "continuous diffusion of oxygen" OR CDO OR "cyclical topical wound oxygen" OR TWO2 OR "local oxygen" OR "oxygen therapy" OR "ozone therapy" OR ozone OR "cold atmospheric plasma" OR "cold plasma" OR "nitric oxide"):ti,ab,kw  #18 #5 OR #6 OR #7 OR #8 OR #9 OR #10 OR #11 OR #12 OR #13 OR #14 OR #15 OR #16 OR #17  #19 #4 AND #18  #20 (randomized OR randomised OR randomly OR placebo OR trial OR "controlled trial" OR "randomized controlled trial" OR "randomised controlled trial"):ti,ab,kw  #21 #19 AND #20  1201 |
| **Embase** | #1 diabetic feet'/exp OR 'diabetic feet' OR 'diabetic foot disease'/exp OR 'diabetic foot disease' OR 'diabetic foot syndrome'/exp OR 'diabetic foot syndrome' OR 'diabetic foot'/exp OR 'diabetic foot'  #2 'thrombocyte rich plasma'/exp OR 'platelet rich plasma' OR 'platelet-rich plasma' OR 'thrombocyte rich plasma'  #3 'growth factor'/exp OR 'growing factor' OR 'growth factor, epithelial cell' OR 'growth factor'  #4 'artificial skin'/exp OR 'dermal substitute' OR 'integra (artificial skin)' OR 'omnigraft' OR 'orcel' OR 'skin substitute' OR 'skin, artificial' OR 'skin, synthetic' OR 'stratagraft' OR 'synthetic skin' OR 'synthetic skin graft' OR 'vitro-skin n19-5x' OR 'artificial skin'  #5 'wound dressing'/exp OR 'adaptic (wound dressing)' OR 'adaptic touch' OR 'algisite' OR 'amd telfa' OR 'aquacel' OR 'askina calgitrol' OR 'askina sorb' OR 'atrauman' OR 'autologel' OR 'biobrane' OR 'biostep' OR 'curasorb' OR 'cutilin' OR 'cutimed sorbact' OR 'dressing, wound' OR 'drymax' OR 'eclypse (wound dressing)' OR 'excellagen' OR 'external pledget' OR 'flivasorb' OR 'graftskin' OR 'hemcon' OR 'hemcon bandage pro' OR 'hemcon dental dressing pro' OR 'hemcon nasal plug' OR 'hemcon strip pro' OR 'inflammatory skin disorder dressing' OR 'jaloskin' OR 'kerrafoam simple' OR 'kerramax' OR 'leukomed' OR 'leukomed sorbact' OR 'medihoney' OR 'non-sterile medicated wound dressing kit' OR 'oasis (wound dressing)' OR 'opsite (wound dressing)' OR 'primapore' OR 'promogran' OR 'quickclot acs' OR 'seasorb' OR 'silvercel' OR 'sorbion' OR 'steri-strips' OR 'sterile medicated wound dressing kit' OR 'sterile non-medicated wound dressing kit' OR 'stratasorb' OR 'suprathel' OR 'surfasoft' OR 'telfa' OR 'tielle' OR 'veloderm' OR 'wound dressing agent' OR 'wound dressing kit' OR 'wound dressing kit, medicated, non-sterile' OR 'wound dressing kit, medicated, sterile' OR 'wound dressing kit, non-medicated, sterile' OR 'zetuvit' OR 'wound dressing'  #6 'vacuum assisted closure'/exp OR 'negative pressure therapy' OR 'negative pressure wound closure' OR 'negative pressure wound therapy' OR 'negative-pressure wound therapy' OR 'npwt (negative pressure wound therapy)' OR 'topical negative pressure' OR 'vacuum assisted closure (vac) therapy' OR 'vacuum assisted closure therapy' OR 'vacuum assisted wound closure' OR 'vacuum therapy' OR 'vacuum wound closure' OR 'vacuum assisted closure'  #7 'oxygen therapy'/exp OR 'o2 administration' OR 'o2 therapy' OR 'oxygen administration' OR 'oxygen inhalation therapy' OR 'oxygen insufflation' OR 'oxygen treatment' OR 'oxygen therapy'  #8 'gel'/exp OR 'gel matrix' OR 'gelcosponge' OR 'gels' OR 'haven gel' OR 'hydraulic gel' OR 'hydrocarbon gel' OR 'hydron gel' OR 'oxygel' OR 'gel'  #9 #2 OR #3 OR #4 OR #5 OR #6 OR #7 OR #8  #10 #1 AND #9  #11 randomized AND controlled AND trial  #12 #10 AND #11 |

| **First author** | **year** | **Country** | **Participant ratio** | **Sex distribution** | **Mean age（years）** | **Treatment duration（week）** | **Intervention in the experimental group** | **Control group intervention** | **Wound area(cm²)** |
| --- | --- | --- | --- | --- | --- | --- | --- | --- | --- |
| Marwa Ahmed[1] | 2017 | Egypt | 28/28 | 38/18 | 43.2± 18.2/49.8±15.4 | 12 | Autologous PRP  gel application | Antiseptic ointment ( povidone iodine) dressing | 6.24±0.9/5.72±0.8 |
| Anil Bhansali[2] | 2009 | India | 10/10 | 12/8 | 51.7±13.6/49.5±8.8 | 20 | recombinant human platelet-derived growth factor-BB | Standard Wound Care | 18.1±15.9/11.1±9.3 |
| Peter Blume[3] | 2011 | US | 72/33/19 | 90/34 | 57.9±10.9/56.2±12/54.8±12.3 | 12 | E1-deleted adenovirus serotype 5 encoding human platelet-derived growth factor-B | Formulated Collagen Gel | 3.1±1.7/2.9±1.1/2.8±1.3 |
| Peter A. Blume[4] | 2008 | US | 169/166 | 263/72 | 58±12/59±12 | 16 | Negative Pressure Wound Therapy | Advanced Moist Wound Therapy | 13.5±18.2/11.0±12.7 |
| Chen-Yu Chen[5] | 2017 | Taiwan, China | 20/18 | 21/17 | 64.3±13/60.8±7.2 | 4 | hyperbaric oxygen therapy | Standard wound care with adjunctive hyperbaric oxygen therapy |  |
| Lawrence A. DiDomenico[6] | 2018 | US | 40/40 | 54/26 | 60.1±11.77/51±10.66 | 12 | Dehydrated Human Amniotic Chorionic Allograft | Standard of Care | 2.1±1.46/3.1±3.58 |
| Vickie R. Driver[7] | 2006 | US | 19/21 | 32/8 | 58.3±9.7/55.9±8.1 | 12 | Autologous platelet-rich plasma | Saline gel dressing | 3.4±4.5/3.6±4.0 |
| Lan Li[8] | 2018 | China | 59/58 | 75/42 | 61.4±13.1/64.1±9.4 | 12 | Autologous platelet-rich gel | Standard treatment | 4.1±6.4/2.9±6.25 |
| Ahmed Elsaid[9] | 2019 | Egypt | 12/12 | 14/10 | 54.7±6.6/55.6±6.5 | 20 | Dressing with platelet-rich plasma gel | Regular dressing with normal saline | 24.58±1.5/15.16±2.4 |
| Arife Polat Duzgun[10] | 2008 | Turkey | 50/50 | 64/36 | 58.1±11.03/63±9.15 | 4 | hyperbaric oxygen therapy | standard therapy |  |
| William J. Ennis[11] | 2005 | US | 27/28 | 28/27 |  | 12 | ultrasound therapy | sham treatment |  |
| Ezio Faglia[12] | 1996 | Italy | 35/33 | 48/20 | 61.7±10.4/65.6±9.1 | 8 | systemic hyperbaric oxygen therapy | Comprehensive protocol |  |
| Ludwik Fedorko[13] | 2016 | Canada | 49/54 | 69/34 | 61±12/62±12 | 6 | hyperbaric oxygen therapy | Sham (Placebo) Therapy | 3.8±4.8/3.6±5.7 |
| José I. Fernández-Montequin[14] | 2009 | Cuba | 53/48/48 | 76/73 | 63±10.37/65.5±11.85/64±14.1 | 8 | Recombinant human epidermal growth factor | Placebo Therapy | 28.5±24/20.1±17/21.8±19.1 |
| Robert G. Frykberg[15] | 2019 | multicenter | 36/37 | 63/10 | 64.6±10.3/61.9±9.5 | 12 | cyclical pressurized Topical Wound Oxygen therapy | sham control therapy | 3.02±2.66/3.22±2.54 |
| Fran Game[16] | 2021 | UK | 15/15 | 25/6 | 62.8±9.21/57±10.39 | 12 | dried human amnion derived membrane | standard care | 0.62±0.57/0.68±0.7 |
| Ramiro Gomez-Villa[17] | 2014 | Mexico | 15/16 | 21/10 | 62.1±12.8/55.1±10.6 | 8 | Recombinant human epidermal growth factor | Placebo Therapy | 19.2±15.7/11.9±11.8 |
| Ajay Gupta[18] | 2021 | India | 30/30 | 41/19 | 56.03±9.6/55.76±10.2 | 6 | freshly prepared Platelet-Rich Plasma | Normal saline dressing | 5.22±3.82/4.96±2.89 |
| Seung-Kyu Han[19] | 2010 | South Korea | 26/26 | 29/23 | 66.5±7.5/68.4±8.7 | 8 | processed lipoaspirate cell | Control treatment | 4.3±2.1/4.0±2.1 |
| Shumin He[20] | 2021 | China | 40/40/40 | 72/48 | 63.5±10.1/63.1±9.3/62.7±7.4 | 8 | Continuous diffusion of oxygen | moist wound dressing | 35.35±20.83/42.07±38.55/39.59±23.36 |
| El Mahdy Hossam[21] | 2021 | Egypt | 40/40 | 62/18 | 54.9±2.37/54.8±3.9 | 6 | autologous platelet-rich plasma | standard wound care | 15.15±5.64/14.5±5.57 |
| Shyam S. Jaiswal[22] | 2010 | India | 25/25 | 42/8 | 56.2±11.34/49.92±18.89 | 10 | recombinant human platelet-derived growth factor | Topical application of a placebo gel | 29.96±3.50/26.5±2.51 |
| S.M. Jeppesen[23] | 2016 | Denmark | 11/12 | 16/7 | 65.3±12.9/67.8±9.7 | 3 | Extracorporeal shockwave therapy | Standard care alone | 2.34±1.66/2.37±0.93 |
| O. Karatepe[24] | 2011 | Turkey | 30/37 | 19/48 | 68.5±11.1/66.3±12.6 | 4 | Vacuum Assisted Closure | Standard wound care | 35.7±6.4/29.7±5.2 |
| Laurence Kessler[25] | 2003 | France | 14/13 | 19/8 | 60.2±9.7/67.6±10.5 | 2 | Hyperbaric Oxygenation Therapy | Standard treatment | 2.31±2.18/2.82±2.43 |
| Sachin Khandelwal[26] | 2013 | India | 20/20/20 | 32/28 | 45±7.57/43.8±9.4/43.35±8.1 | 10 | Hyperbaric Oxygen Therapy/Recombinant human platelet derived growth factor | Antiseptic Dressings | 9.9±5.59/14.91±6.23/19.26±11.32 |
| Atit Kumar[27] | 2020 | India | 28/26 | 39/15 | 58.4±10.1/56.9±11.1 | 6 | hyperbaric oxygen therapy | Standard treatment alone | 2.9±1.5/3.0±2.8 |
| Lawrence A. Lavery[28] | 2014 | US | 50/47 | 68/29 | 55.5±11.5/55.1±12 | 12 | Human viable wound matrix | Standard wound care | 3.41±3.23/3.93±3.22 |
| Nasser Malekpour Alamdari[29] | 2021 | Iran | 43/47 | 56/34 | 56.3±7.1/56.7±7.2 | 3 | Platelet-rich plasma | Conventional dressing therapy |  |
| Haraesh Maranna[30] | 2021 | India | 22/23 | 33/12 | 50.23±10.52/49±10.14 | 2 | Negative Pressure Wound Therapy | Conventional saline dressings | 48.45±17.42/47.3±17 |
| Nasser Malekpour Alamdari[31] | 2021 | Iran | 30/30 | 33/27 | 70.31±5.92/71.8±6.32 | 12 | Negative Pressure Wound Therapy | Conventional dressing | 15.07±2.92/14.09±2.81 |
| Biagio Moretti[32] | 2009 | Italy | 15/15 | 16/14 | 56.2±4.9/56.8±7.5 | 20 | extracorporeal shock wave therapy | Standard care alone | 2.98±1.29/2.45±1.01 |
| PS Nain[33] | 2011 | India | 15/15 | 25/5 | 61.33±7.63/55.4±11.54 | 8 | Negative Pressure Wound Therapy | Conventionalsaline-moistened gauze dressing |  |
| MQ. Niederauer[34] | 2017 | US | 50/50 | 79/21 | 57.5±10.9/59.1±13.3 | 12 | continuous diffusion of oxygen | Sham therapy | 3.4±1.5/3.9±2.0 |
| Mark Q. Niederauer[35] | 2018 | US | 74/72 | 113/33 | 56.1±10.1/56.6±14.4 | 12 | continuous diffusion of oxygen | Placebo arm | 3.54±1.68/3.89±2.02 |
| Mohammed T.A. Omar[36] | 2014 | Saudi Arabia | 19/19 | 27/11 | 56.59±7.35/57±5.39 | 8 | Extracorporeal shock wave therapy | Standard wound care | 7.89±2.97/8.62±3.47 |
| Yasser A Orban[37] | 2022 | Egypt | 36/36 | 41/31 | 56.03±8.39/58.69±6.68 | 20 | Platelet-rich Plasma | Conventional dressing | 3.33±1.31/3.23±1.2 |
| Kwang Hwan Park[38] | 2018 | South Korea | 82/85 | 104/63 | 56.52±12.71/59.31±12.64 | 12 | recombinant human epidermal growth factor | saline spray | 2.8±3.72/2.35±2.69 |
| Ashu Rastogi[39] | 2019 | India | 34/24 |  | 52.5±7.2/51.2±7.3 | 4 | low-frequency airborne ultrasound therapy | Sham therapy | 11.3±8.2/14.8±13.8 |
| Shimaa Elhossieny Salama[40] | 2019 | Egypt | 15/15 | 22/8 | 55.1±7.5/57.7±6.7 | 8 | Hyperbaric oxygen therapy | Conventional treatment alone | 7.5±8.5/8.0±9.25 |
| Dorthe Seidel[41] | 2022 | Germany | 44/110 | 113/41 | 66.5±11/67.8±10.4 | 16 | Negative pressure wound therapy | Standard moist wound care | 9.64±13.92/8.78±12.66 |
| Dorthe Seidel[42] | 2020 | Germany | 171/174 | 267/78 | 67.6±12.3/68.1±11.5 | 16 | Negative pressure wound therapy | Standard moist wound care | 11.01±25.43/11.41±32.47 |
| Thomas E Serena[43] | 2021 | US | 81/64 | 107/37 | 64.2±14.15/62.69±12.56 | 12 | topical oxygen therapy | Standard care | 2.86±2.93/3.47±4.12 |
| Thomas E Serena[44] | 2019 | US | 38/38 | 59/17 | 59.2±7.61/59.6±10.72 | 16 | hypothermically stored amniotic membrane | Standard of care | 3.12±3.86/3.33±4.62 |
| Shailendra Pal Singh[45] | 2018 | India | 29/26 | 34/21 | 53.76±10.38/55.69±10.35 | 4 | Treatment with platelet-rich plasma | Standard therapy |  |
| Sanjeev Singla[46] | 2014 | India | 25/25 | 44/6 | 58.8±27.5/55.84±27.5 | 8 | recombinant human epidermal growth factor | Betadine dressing | 19.56/21.20 |
| Robert Snyder[47] | 2018 | multicenter | 172/164 | 269/67 | 59.9±10/56.4±9.9 | 12 | extracorporeal shockwave therapy | sham therapy | 3.55±3.1/3.16±2.5 |
| William Tettelbach[48] | 2018 | US | 54/56 | 80/30 | 57.4±10.6/57.1±10.5 | 12 | dehydrated human amnion/chorion membrane | Standard wound care alone | 3.2±2.8/3.9±3.8 |
| William Tettelbach[49] | 2018 | US | 101/54 | 126/29 | 58.3±10.9/56.3±10.2 | 12 | dehydrated human amnion/chorion membrane | Standard wound care alone | 2.6±2.2/2.8±2.6 |
| Patricia Thompson[50] | 2019 | US | 7/6 | 11/2 | 58.5±12.96/55.17±18.32 | 12 | human amniotic allograft | standard wound care | 1.54±3.61/2.78±5.69 |
| Erdal Uzun[51] | 2020 | Turkey | 10/10 | 12/8 | 57.5±8.4/57.2±4.5 | 8 | allogeneic adipose-derived mesenchymal stem cells | Standard diabetic wound care alone | 23.5±5.6/25.8±5.4 |
| Venkat N. Vangaveti[52] | 2023 | Australia | 25/23 | 34/14 | 62±13/62±13.5 | 6 | Extracorporeal shockwave therapy | Standard of care | 0.7±1.05/0.48±1.94 |
| Vijay Viswanathan[53] | 2019 | India | 27/23 | 27/23 | 57.9±9.6/55±6.8 | 8 | recombinant human epidermal growth factor | Alternative placebo treatment. | 9.1±9.5/8.4±7.9 |
| Ching-Jen Wang[54] | 2011 | Taiwan, China | 44/40 |  | 60.51±13.97/62.45±13.95 | 3 | Extracorporeal Shockwave Therapy | Hyperbaric Oxygen Therapy | 4±3.75/7±5 |
| T. Jeffery Wieman[55] | 1998 | US | 132/123/127 | 255/127 | 58±11.3/57±11.5/58±11.8 | 20 | recombinant human platelet-derived growth factor-BB | placebo gel | 2.6±2.69/2.6±3.41/2.8±4.14 |
| Charles M Zelen[56] | 2013 | US | 13/12 | 16/9 | 56.4±14.7/61.7±10.3 | 12 | human amniotic membrane allograft | Standard of care | 2.6±1.9/3.4±2.9 |
| Charles M Zelen[57] | 2016 | US | 33/32/35 | 55/45 | 63.8±11.86/63.3±12.25/60.6 ±11.55 | 12 | bioengineered skin substitute/dehydrated human amnion/chorion membrane | Standard wound care | 2.7±2.75/2.6±2.97/3.1±3.17 |
| Yu-Yao Huang[58] | 2021 | multicenter | 122/114 | 175/61 | 57.4±10.6/56.6±11.3 | 16 | Topical application of ON101 cream | absorbent dressing | 5±4.4/5.1±4.7 |
| Jui-Yung Yang[59] | 2022 | Taiwan, China | 90/42 | 81/51 | 62±14.24/64±13.38 | 12 | ENERGI-F703 gel | vehicle gel | 3.78±6.92/3.47±7.26 |
| Raúl Loera Valencia[60] | 2022 | Mexico | 16/10 | 19/7 | 48.38±10.18/45.4±11.07 | 10 | Calcium alginate dressings with ZnO nanoparticles | Calcium alginate dressings without nanoparticles | 16.71±18.86/29.01±32.16 |
| Saher Hamed[61] | 2019 | multicenter | 5/5 | 6/4 | 66±9.4/69.6±7.8 | 12 | topical RMD-G1 | Standard of care | 5±4.9/7.5±5.5 |
| Giuseppe Gallelli[62] | 2019 | Italy | 28/28 | 28/28 | 69.3±9.2/67.9±8.4 | 8 | nano-hydrogel (0.2%) embedded with quercetin and oleic acid | hyaluronic acid (0.2%) |  |
| Peter P. Balingit[63] | 2012 | US | 54/24 | 60/18 | 56.3±10.35/53.1±12.47/56.8 ±12.77 | 4 | NorLeu3-A(1-7) in hydroxyethyl cellulose vehicle | placebo therapy | 2.4±1.285/1.91±0.971/1.94 ±1.218 |
| Kyung-Chul Moon[64] | 2019 | South Korea | 22/17 | 27/12 | 59.9±13.3/68.4±9.9 | 12 | ALLO-ASC-Sheet | Application of polyurethane film | 2.0±0.9/2.8±2.0 |
| Manar Moustafa[65] | 2007 | UK | 7/5 | 9/3 | 52.4 | 12 | autologous keratinocytes | placebo therapy |  |
| Luis A. B. Tonaco[66] | 2018 | Brazil | 27/23 | 25/25 |  | 16 | 0.1% P1G10 | Hydrogel treatment |  |
| Aida Rezaei-Nejad[67] | 2023 | Iran | 9/9 | 8/10 | 58.19±4.49/60.24±1.83 | 9 | lyophilized amniotic membrane gel | Standard care and a gelatin scaffold | 4.04/3.97 |
| Saher Hamed[68] | 2021 | Israel | 10/8 | 13/5 | 64.3±11.7/66.5±8.1 | 12 | erythropoietin | Standard care | 5.0±4.1/6.1±4.7 |
| Ashu Rastogi[69] | 2023 | India | 76/76 | 122/54 | 56.5±9.0/56.1±9.0 | 12 | esmolol, 14%, gel | Standard care | 4.59±2.77/4.75±2.9 |
| Janelle Yu[70] | 2016 | Canada | 10/10 | 17/3 | 57±9.5/58±9.5 | 8 | topical oxygen | Standard care | 1.37±0.95/1.68±1.31 |
| Le Ma[71] | 2013 | China | 18/18 | 23/13 | 59.8±6.5/60.4±5.6 | 2 | hyperbaric oxygen | Standard care alone | 4.21±0.99/4.35±1.04 |
| Oliver J. Smith[72] | 2020 | UK | 6/6/6 | 15/3 | 55.2/60.2/57.5 | 12 | Fat grafting/Fat grafting with platelet-rich plasma | Podiatry care | 1.3±3.2/3.1±4.3/6.4±4.0 |
| Stephen A. Brigido[73] | 2006 | US | 14/14 |  | 61.43±7.18/66.21±4.37 | 16 | Graftjacket tissue matrix | CuraSol wound gel |  |
| Vickie R. Driver[74] | 2015 | US | 154/153 | 232/75 | 55.8±10.6/57.3±10.1 | 16 | Integra Dermal Regeneration Template | Standard of care | 3.53±2.5/3.65±2.7 |
| Jodi Walters[75] | 2016 | US | 53/56 |  | 58±13.1/57.1±10.9 | 16 | acellular dermal matrix | Conventional wound care | 3.6±4.2/3.1±3.0 |
| Alexander Reyzelman[76] | 2009 | US | 47/39 |  | 55.4±9.6/58.9±11.6 | 12 | human acellular dermal regenerative tissue matrix graft | Standard-of-care wound management | 3.6±4.3/5.1±4.8 |
| Charles M Zelen[77] | 2017 | US | 20/20 | 28/12 | 61.5±10.85/57.1±10.65 | 12 | human reticular acellular dermal matrix | Standard of care | 4.7±5.24/2.7±2.26 |
| Charles M Zelen[78] | 2018 | US | 40/40 | 52/28 | 59±12/62±13 | 12 | human reticular acellular dermal matrix | Standard of care | 3.2±4.0/2.7±2.4 |
| Haiyun Gu[79] | 2025 | China | 204/246 | 353/97 | 60±10/60±10 | 16 | Negative-pressure wound therapy | Advanced moist wound therapy | 13.1±7.0/13.4±6.0 |
| Arash Mohammadi Tofigh[80] | 2022 | Iran | 81/81/81 | 139/104 | 57.9±5.8/55.8±5.6/60.2±5.2 | 12 | dehydrated amnion powder/platelet-derived growth factor gel | standard method | 2.75±2.22/2.68±1.76/3.29±1.51 |
| Paul M. Glat[81] | 2022 | US | 50/50 | 53/47 | 61.2±12.55/60±10.99 | 12 | Bioactive split thickness skin allograft | Standard of Care | 4.2±7.8/3.9±5.22 |
| Eric J. Lullove[82] | 2022 | US | 46/48 |  |  | 12 | fish skin graft | Standard of care |  |
| David G. Armstrong[83] | 2022 | US | 20/20 | 20/20 | 61.0±13.81/64.3±9.32 | 12 | resorbable glass microfiber matrix | Standard of care alone | 3.5±2.07/5.2±4.67 |
| David G. Armstrong[84] | 2022 | US | 20/20 | 25/15 | 59.3±13.35/66.5±11.26 | 12 | purified reconstituted bilayer matrix | Standard of care | 2.5±2.16/3.5±2.85 |

**Table S3** When multiple reports described the same or overlapping randomized trial population, data were extracted at the trial level for each outcome to avoid duplicate counting. Active comparators, sham/placebo controls, and enhanced dressing controls were classified according to whether both arms received comparable standard of care; this classification should be considered when interpreting transitivity and indirectness.

[1] M. Ahmed, S.A. Reffat, A. Hassan, and F.J.A.o.v.s. Eskander, Platelet-rich plasma for the treatment of clean diabetic foot ulcers. 38 (2017) 206-211.

[2] A. Bhansali, S. Venkatesh, P. Dutta, M. Dhillon, S. Das, A.J.D.R. Agrawal, and C. Practice, Which is the better option: recombinant human PDGF-BB 0.01% gel or standard wound care, in diabetic neuropathic large plantar ulcers off-loaded by a customized contact cast? 83 (2009) e13-e16.

[3] P. Blume, V.R. Driver, A.J. Tallis, R.S. Kirsner, R. Kroeker, W.G. Payne, S. Wali, W. Marston, C. Dove, R.L.J.W.r. Engler, and regeneration, Formulated collagen gel accelerates healing rate immediately after application in patients with diabetic neuropathic foot ulcers. 19 (2011) 302-308.

[4] P.A. Blume, J. Walters, W. Payne, J. Ayala, and J.J.D.c. Lantis, Comparison of negative pressure wound therapy using vacuum-assisted closure with advanced moist wound therapy in the treatment of diabetic foot ulcers: a multicenter randomized controlled trial. 31 (2008) 631-636.

[5] C.-Y. Chen, R.-W. Wu, M.-C. Hsu, C.-J. Hsieh, M.-C.J.J.o.W.O. Chou, and C. Nursing, Adjunctive hyperbaric oxygen therapy for healing of chronic diabetic foot ulcers: a randomized controlled trial. 44 (2017) 536-545.

[6] L.A. DiDomenico, D.P. Orgill, R.D. Galiano, T.E. Serena, M.J. Carter, J.P. Kaufman, N.J. Young, A.M. Jacobs, and C.M.J.I.W.J. Zelen, Use of an aseptically processed, dehydrated human amnion and chorion membrane improves likelihood and rate of healing in chronic diabetic foot ulcers: a prospective, randomised, multi‐centre clinical trial in 80 patients. 15 (2018) 950-957.

[7] V.R. Driver, J. Hanft, C.P. Fylling, and J.M.J.O.W.M. Beriou, A prospective, randomized, controlled trial of autologous platelet-rich plasma gel for the treatment of diabetic foot ulcers. 52 (2006) 68.

[8] L. Li, D. Chen, C. Wang, N. Yuan, Y. Wang, L. He, Y. Yang, L. Chen, G. Liu, X.J.W.R. Li, and Regeneration, Autologous platelet‐rich gel for treatment of diabetic chronic refractory cutaneous ulcers: a prospective, randomized clinical trial. 23 (2015) 495-505.

[9] A. Elsaid, M. El‐Said, S. Emile, M. Youssef, W. Khafagy, and A.J.W.j.o.s. Elshobaky, Randomized controlled trial on autologous platelet‐rich plasma versus saline dressing in treatment of non‐healing diabetic foot ulcers. 44 (2020) 1294-1301.

[10] A.P. Duzgun, H.Z. Satır, O. Ozozan, B. Saylam, B. Kulah, F.J.T.J.o.f. Coskun, and a. surgery, Effect of hyperbaric oxygen therapy on healing of diabetic foot ulcers. 47 (2008) 515-519.

[11] W.J. Ennis, P. Formann, N. Mozen, J. Massey, T. Conner-Kerr, and P.J.O.w.m. Meneses, Ultrasound therapy for recalcitrant diabetic foot ulcers: results of a randomized, double-blind, controlled, multicenter study. 51 (2005) 24.

[12] E. Faglia, F. Favales, A. Aldeghi, P. Calia, A. Quarantiello, G. Oriani, M. Michael, P. Campagnoli, and A.J.D.c. Morabito, Adjunctive systemic hyperbaric oxygen therapy in treatment of severe prevalently ischemic diabetic foot ulcer: a randomized study. 19 (1996) 1338-1343.

[13] L. Fedorko, J.M. Bowen, W. Jones, G. Oreopoulos, R. Goeree, R.B. Hopkins, and D.J.J.D.c. O’Reilly, Hyperbaric oxygen therapy does not reduce indications for amputation in patients with diabetes with nonhealing ulcers of the lower limb: a prospective, double-blind, randomized controlled clinical trial. 39 (2016) 392-399.

[14] J.I. Fernández‐Montequín, C.M. Valenzuela‐Silva, O.G. Díaz, W. Savigne, N. Sancho‐Soutelo, F. Rivero‐Fernández, P. Sánchez‐Penton, L. Morejón‐Vega, H. Artaza‐Sanz, and A.J.I.w.j. García‐Herrera, Intra‐lesional injections of recombinant human epidermal growth factor promote granulation and healing in advanced diabetic foot ulcers: multicenter, randomised, placebo‐controlled, double‐blind study. 6 (2009) 432-443.

[15] R. Frykberg, Commentary on A multinational, multicenter, randomized, double-blinded, placebo-controlled trial to evaluate the efficacy of cyclical topical wound oxygen therapy (TWO2) in the treatment of chronic diabetic foot ulcers: The TWO2 study. (2019).

[16] F. Game, K. Gray, D. Davis, R. Sherman, K. Chokkalingam, Z. Connan, A. Fakis, and M.J.I.W.J. Jones, The effectiveness of a new dried human amnion derived membrane in addition to standard care in treating diabetic foot ulcers: a patient and assessor blind, randomised controlled pilot study. 18 (2021) 692-700.

[17] R. Gomez‐Villa, F. Aguilar‐Rebolledo, A. Lozano‐Platonoff, J.M. Teran‐Soto, M.R. Fabian‐Victoriano, N.S. Kresch‐Tronik, X. Garrido‐Espíndola, A. Garcia‐Solis, A. Bondani‐Guasti, G.J.W.R. Bierzwinsky‐Sneider, and Regeneration, Efficacy of intralesional recombinant human epidermal growth factor in diabetic foot ulcers in M exican patients: A randomized double‐blinded controlled trial. 22 (2014) 497-503.

[18] A. Gupta, C. Channaveera, S. Sethi, S. Ranga, and V.J.J.o.t.A.P.M.A. Anand, Efficacy of intralesional platelet-rich plasma in diabetic foot ulcer. 111 (2021).

[19] S.K. Han, H.R. Kim, W.K.J.W.r. Kim, and regeneration, The treatment of diabetic foot ulcers with uncultured, processed lipoaspirate cells: a pilot study. 18 (2010) 342-348.

[20] S. He, C. Liang, C. Yi, M.J.D.r. Wu, and c. practice, Therapeutic effect of continuous diffusion of oxygen therapy combined with traditional moist wound dressing therapy in the treatment of diabetic foot ulcers. 174 (2021) 108743.

[21] A.H. Alserr, C.N. Antonopoulos, A. Zaki, and W.J.A.o.v.s. Eldaly, Autologous platelet rich plasma promotes the healing of non-ischemic diabetic foot ulcers. A randomized controlled trial. 82 (2022) 165-171.

[22] S.S. Jaiswal, R. Gambhir, A. Agrawal, and S.J.I.J.o.S. Harish, Efficacy of topical recombinant human platelet derived growth factor on wound healing in patients with chronic diabetic lower limb ulcers. 72 (2010) 27-31.

[23] S. Jeppesen, K. Yderstraede, B. Rasmussen, M. Hanna, and L.J.J.o.w.c. Lund, Extracorporeal shockwave therapy in the treatment of chronic diabetic foot ulcers: a prospective randomised trial. 25 (2016) 641-649.

[24] O. Karatepe, I. Eken, E. Acet, O. Unal, M. Mert, B. Koc, S. Karahan, U. Filizcan, M. Ugurlucan, and M.J.A.C.B. Aksoy, Vacuum assisted closure improves the quality of life in patients with diabetic foot. 111 (2011) 298-302.

[25] L. Kessler, P. Bilbault, F. ORTega, C. Grasso, R. Passemard, D. Stephan, M. Pinget, and F.J.D.c. Schneider, Hyperbaric oxygenation accelerates the healing rate of nonischemic chronic diabetic foot ulcers: a prospective randomized study. 26 (2003) 2378-2382.

[26] S. Khandelwal, P. Chaudhary, D.D. Poddar, N. Saxena, R.A. Singh, U.C.J.C. Biswal, and practice, Comparative study of different treatment options of grade III and IV diabetic foot ulcers to reduce the incidence of amputations. 3 (2013) e9.

[27] A. Kumar, U. Shukla, T. Prabhakar, and D.J.J.o.A.C.P. Srivastava, Hyperbaric oxygen therapy as an adjuvant to standard therapy in the treatment of diabetic foot ulcers. 36 (2020) 213-218.

[28] L.A. Lavery, J. Fulmer, K.A. Shebetka, M. Regulski, D. Vayser, D. Fried, H. Kashefsky, T.M. Owings, J. Nadarajah, and G.D.F.U.S.G.J.I.w. journal, The efficacy and safety of Grafix® for the treatment of chronic diabetic foot ulcers: results of a multi‐centre, controlled, randomised, blinded, clinical trial. 11 (2014) 554-560.

[29] N.M. Alamdari, A. Shafiee, A. Mirmohseni, S.J.D. Besharat, M.S.C. Research, and Reviews, Evaluation of the efficacy of platelet-rich plasma on healing of clean diabetic foot ulcers: A randomized clinical trial in Tehran, Iran. 15 (2021) 621-626.

[30] H. Maranna, P. Lal, A. Mishra, L. Bains, G. Sawant, R. Bhatia, P. Kumar, M.Y.J.D. Beg, m.s.c. research, and reviews, Negative pressure wound therapy in grade 1 and 2 diabetic foot ulcers: A randomized controlled study. 15 (2021) 365-371.

[31] N. Malekpour Alamdari, B. Mehraneroodi, B. Gholizadeh, A. Zeinalpour, P. Safe, and S.J.I.J.o.D.i.D.C. Besharat, The efficacy of negative pressure wound therapy compared with conventional dressing in treating infected diabetic foot ulcers: a randomized controlled trial. 41 (2021) 664-668.

[32] B. Moretti, A. Notarnicola, G. Maggio, L. Moretti, M. Pascone, S. Tafuri, and V.J.B.m.d. Patella, The management of neuropathic ulcers of the foot in diabetes by shock wave therapy. 10 (2009) 54.

[33] P.S. Nain, S.K. Uppal, R. Garg, K. Bajaj, S.J.J.o.s.t. Garg, and c. report, Role of negative pressure wound therapy in healing of diabetic foot ulcers. 3 (2011) 17.

[34] M.Q. Niederauer, J.E. Michalek, D.G.J.J.o.d.s. Armstrong, and technology, A prospective, randomized, double-blind multicenter study comparing continuous diffusion of oxygen therapy to sham therapy in the treatment of diabetic foot ulcers. 11 (2017) 883-891.

[35] M.Q. Niederauer, J.E. Michalek, Q. Liu, K.K. Papas, L.A. Lavery, and D.G.J.J.o.w.c. Armstrong, Continuous diffusion of oxygen improves diabetic foot ulcer healing when compared with a placebo control: a randomised, double-blind, multicentre study. 27 (2018) S30-S45.

[36] M.T. Omar, A. Alghadir, K.K. Al-Wahhabi, A.B.J.D.r. Al-Askar, and c. practice, Efficacy of shock wave therapy on chronic diabetic foot ulcer: a single-blinded randomized controlled clinical trial. 106 (2014) 548-554.

[37] Y.A. Orban, M.A. Soliman, Y.H. Hegab, M.M.J.W.A.C.o.C.R. Alkilany, and Practice, Autologous platelet-rich plasma vs conventional dressing in the management of chronic diabetic foot ulcers. 33 (2022) 36-42.

[38] K.H. Park, S.H. Han, J.P. Hong, S.-K. Han, D.-H. Lee, B.S. Kim, J.H. Ahn, J.W.J.D.r. Lee, and c. practice, Topical epidermal growth factor spray for the treatment of chronic diabetic foot ulcers: A phase III multicenter, double-blind, randomized, placebo-controlled trial. 142 (2018) 335-344.

[39] A. Rastogi, A. Bhansali, and S.J.T.I.J.o.L.E.W. Ramachandran, Efficacy and safety of low-frequency, noncontact airborne ultrasound therapy (Glybetac) for neuropathic diabetic foot ulcers: a randomized, double-blind, sham-control study. 18 (2019) 81-88.

[40] S.E. Salama, A.E. Eldeeb, A.H. Elbarbary, and S.E.J.T.i.j.o.l.e.w. Abdelghany, Adjuvant hyperbaric oxygen therapy enhances healing of nonischemic diabetic foot ulcers compared with standard wound care alone. 18 (2019) 75-80.

[41] D. Seidel, R. Lefering, D.s.g.S.M.L.H.W.G.M.P.H.D.W.-R.W.S.K.H.M.R.G.K.T.Z.K.J.J.o. foot, and a. research, NPWT resource use compared with standard moist wound care in diabetic foot wounds: DiaFu randomized clinical trial results. 15 (2022) 72.

[42] D. Seidel, M. Storck, H. Lawall, G. Wozniak, P. Mauckner, D. Hochlenert, W. Wetzel-Roth, K. Sondern, M. Hahn, and G. Rothenaicher, Negative pressure wound therapy compared with standard moist wound care on diabetic foot ulcers in real-life clinical practice: Results of the German DiaFu-RCT. BMJ Open, 10 (3), e026345, 2020.

[43] T.E. Serena, N.M. Bullock, W. Cole, J. Lantis, L. Li, S. Moore, K. Patel, M. Sabo, N. Wahab, and P.J.J.o.w.c. Price, Topical oxygen therapy in the treatment of diabetic foot ulcers: a multicentre, open, randomised controlled clinical trial. 30 (2021) S7-S14.

[44] T.E. Serena, R. Yaakov, S. Moore, W. Cole, S. Coe, R. Snyder, K. Patel, B. Doner, M.A. Kasper, and R.J.J.o.c.e.r. Hamil, A randomized controlled clinical trial of a hypothermically stored amniotic membrane for use in diabetic foot ulcers. 9 (2020) 23-34.

[45] S.P. Singh, V. Kumar, A. Pandey, P. Pandey, V. Gupta, and R.J.J.o.W.C. Verma, Role of platelet-rich plasma in healing diabetic foot ulcers: a prospective study. 27 (2018) 550-556.

[46] S. Singla, R. Garg, A. Kumar, C.J.J.o.n.s. Gill, biology,, and medicine, Efficacy of topical application of beta urogastrone (recombinant human epidermal growth factor) in Wagner's grade 1 and 2 diabetic foot ulcers: comparative analysis of 50 patients. 5 (2014) 273.

[47] R. Snyder, R. Galiano, P. Mayer, L.C. Rogers, O. Alvarez, and S.T.I.J.J.o.W. Care, Diabetic foot ulcer treatment with focused shockwave therapy: two multicentre, prospective, controlled, double-blinded, randomised phase III clinical trials. 27 (2018) 822-836.

[48] W. Tettelbach, S. Cazzell, A.M. Reyzelman, F. Sigal, J.M. Caporusso, and P.S.J.I.w.j. Agnew, A confirmatory study on the efficacy of dehydrated human amnion/chorion membrane dHACM allograft in the management of diabetic foot ulcers: a prospective, multicentre, randomised, controlled study of 110 patients from 14 wound clinics. 16 (2019) 19-29.

[49] W. Tettelbach, S. Cazzell, F. Sigal, J.M. Caporusso, P.S. Agnew, J. Hanft, and C.J.I.w.j. Dove, A multicentre prospective randomised controlled comparative parallel study of dehydrated human umbilical cord (EpiCord) allograft for the treatment of diabetic foot ulcers. 16 (2019) 122-130.

[50] P. Thompson, D.S. Hanson, D. Langemo, J.J.A.i.s. Anderson, and w. care, Comparing human amniotic allograft and standard wound care when using total contact casting in the treatment of patients with diabetic foot ulcers. 32 (2019) 272-277.

[51] E. Uzun, A. Güney, Z.B. Gönen, Y. Özkul, İ.H. Kafadar, M. Günay, M.J.F. Mutlu, and A. Surgery, Intralesional allogeneic adipose-derived stem cells application in chronic diabetic foot ulcer: Phase I/2 safety study. 27 (2021) 636-642.

[52] V.N. Vangaveti, S. Jhamb, J. Goodall, J. Bulbrook, E. Biros, U.H.J.T.J.o.F. Malabu, and A. Surgery, Extracorporeal shockwave therapy (ESWT) in the management of diabetic foot ulcer: a prospective randomized clinical trial. 62 (2023) 845-849.

[53] V. Viswanathan, U. Juttada, and M.J.T.I.J.o.L.E.W. Babu, Efficacy of recombinant human epidermal growth factor (Regen-D 150) in healing diabetic foot ulcers: a hospital-based randomized controlled trial. 19 (2020) 158-164.

[54] C.-J. Wang, R.-W. Wu, Y.-J.J.D.r. Yang, and c. practice, Treatment of diabetic foot ulcers: a comparative study of extracorporeal shockwave therapy and hyperbaric oxygen therapy. 92 (2011) 187-193.

[55] T.J. Wieman, J.M. Smiell, and Y.J.D.c. Su, Efficacy and safely of a topical gel formulation of recombinant human platelet-derived growth factor-BB (becaplermin) in patients with chronic neuropathic diabetic ulcers: a phase III randomized placebo-controlled double-blind study. 21 (1998) 822-827.

[56] C.M. Zelen, T.E. Serena, G. Denoziere, and D.E.J.I.w.j. Fetterolf, A prospective randomised comparative parallel study of amniotic membrane wound graft in the management of diabetic foot ulcers. 10 (2013) 502-507.

[57] C.M. Zelen, T.E. Serena, L. Gould, L. Le, M.J. Carter, J. Keller, and W.W.J.I.w.j. Li, Treatment of chronic diabetic lower extremity ulcers with advanced therapies: a prospective, randomised, controlled, multi‐centre comparative study examining clinical efficacy and cost. 13 (2016) 272-282.

[58] Y.-Y. Huang, C.-W. Lin, N.-C. Cheng, S.M. Cazzell, H.-H. Chen, K.-F. Huang, K.-Y. Tung, H.-L. Huang, P.-Y. Lin, and C.-K.J.J.n.o. Perng, Effect of a novel macrophage-regulating drug on wound healing in patients with diabetic foot ulcers: a randomized clinical trial. 4 (2021) e2122607.

[59] J.-Y. Yang, C.-C. Chen, S.-C. Chang, J.-T. Yeh, H.-F. Huang, H.-C. Lin, S.-H. Lin, Y.-H. Lin, L.-G. Wei, and T.J.J.E. Liu, ENERGI-F703 gel, as a new topical treatment for diabetic foot and leg ulcers: A multicenter, randomized, double-blind, phase II trial. 51 (2022).

[60] R. Loera-Valencia, R.E. Neira, B.P. Urbina, A. Camacho, R.B.J.B. Galindo, and Pharmacotherapy, Evaluation of the therapeutic efficacy of dressings with ZnO nanoparticles in the treatment of diabetic foot ulcers. 155 (2022) 113708.

[61] S. Hamed, M. Belokopytov, Y. Ullmann, M. Safadi, Y. Stark, A. Shoufani, S. Akita, P.Y. Liu, and L. Teot, Interim results of the remede d'Or study: a multicenter, single-blind, randomized, controlled trial to assess the safety and efficacy of an innovative topical formulation of erythropoietin for treating diabetic foot ulcers, SAGE Publications Sage CA: Los Angeles, CA, 2019.

[62] G. Gallelli, E. Cione, R. Serra, A. Leo, R. Citraro, P. Matricardi, C. Di Meo, F. Bisceglia, M.C. Caroleo, and S.J.I.w.j. Basile, Nano‐hydrogel embedded with quercetin and oleic acid as a new formulation in the treatment of diabetic foot ulcer: A pilot study. 17 (2020) 485-490.

[63] P.P. Balingit, D.G. Armstrong, A.M. Reyzelman, L. Bolton, S.J. Verco, K.E. Rodgers, K.A. Nigh, G.S.J.W.R. DiZerega, and Regeneration, NorLeu 3‐A (1–7) stimulation of diabetic foot ulcer healing: Results of a randomized, parallel‐group, double‐blind, placebo‐controlled phase 2 clinical trial. 20 (2012) 482-490.

[64] K.-C. Moon, H.-S. Suh, K.-B. Kim, S.-K. Han, K.-W. Young, J.-W. Lee, and M.-H.J.D. Kim, Potential of allogeneic adipose-derived stem cell–hydrogel complex for treating diabetic foot ulcers. 68 (2019) 837-846.

[65] M. Moustafa, A.J. Bullock, F.M. Creagh, S. Heller, W. Jeffcoate, F. Game, C. Amery, S. Tesfaye, Z. Ince, and D.B.J.R.m. Haddow, Randomized, controlled, single-blind study on use of autologous keratinocytes on a transfer dressing to treat nonhealing diabetic ulcers. 2 (2007) 887-902.

[66] L.A. Tonaco, F.L. Gomes, G. Velasquez-Melendez, M.T. Lopes, and C.E.J.A.i.T. Salas, The proteolytic fraction from latex of Vasconcellea cundinamarcensis (P1G10) enhances wound healing of diabetic foot ulcers: A double-blind randomized pilot study. 35 (2018) 494-502.

[67] A. Rezaei-Nejad, M.-A. Amirkhani, A. Ebrahimi, S.-M. Ghorani, E. Alamoutifard, M.-A. Nilforoushzadeh, and M.J.T.I.J.o.L.E.W. Mollapour-Sisakht, The therapeutic efficacy of freeze-dried human amniotic membrane allograft gel for diabetic foot ulcers: a phase-1 clinical trial. 25 (2026) 163-173.

[68] S. Hamed, Y. Ullmann, M. Belokopytov, A. Shoufani, H. Kabha, S. Masri, Z. Feldbrin, L. Kogan, D. Kruchevsky, and R.J.R.r. Najjar, Topical erythropoietin accelerates wound closure in patients with diabetic foot ulcers: a prospective, multicenter, single-blind, randomized, controlled trial. 24 (2021) 251-261.

[69] A. Rastogi, S.A. Kulkarni, S. Agarwal, M. Akhtar, S. Arsule, S. Bhamre, D. Bhosle, S. Desai, M. Deshmukh, and K.J.J.N.O. Giriraja, Topical esmolol hydrochloride as a novel treatment modality for diabetic foot ulcers: a phase 3 randomized clinical trial. 6 (2023) e2311509.

[70] J. Yu, S. Lu, A.M. McLaren, J.A. Perry, K.M.J.W.R. Cross, and Regeneration, Topical oxygen therapy results in complete wound healing in diabetic foot ulcers. 24 (2016) 1066-1072.

[71] L. Ma, P. Li, Z. Shi, T. Hou, X. Chen, and J. Du, A prospective, randomized, controlled study of hyperbaric oxygen therapy: effects on healing and oxidative stress of ulcer tissue in patients with a diabetic foot ulcer, 2013.

[72] O.J. Smith, R. Leigh, M. Kanapathy, P. Macneal, G. Jell, N. Hachach‐Haram, H. Mann, and A.J.I.W.J. Mosahebi, Fat grafting and platelet‐rich plasma for the treatment of diabetic foot ulcers: a feasibility‐randomised controlled trial. 17 (2020) 1578-1594.

[73] S.A.J.I.w.j. Brigido, The use of an acellular dermal regenerative tissue matrix in the treatment of lower extremity wounds: a prospective 16‐week pilot study. 3 (2006) 181-187.

[74] V.R. Driver, L.A. Lavery, A.M. Reyzelman, T.G. Dutra, C.R. Dove, S.V. Kotsis, H.M. Kim, K.C.J.W.R. Chung, and Regeneration, A clinical trial of Integra Template for diabetic foot ulcer treatment. 23 (2015) 891-900.

[75] J. Walters, S. Cazzell, H. Pham, D. Vayser, and A.J.E. Reyzelman, Healing rates in a multicenter assessment of a sterile, room temperature, acellular dermal matrix versus conventional care wound management and an active comparator in the treatment of full-thickness diabetic foot ulcers. 16 (2016) e10.

[76] A. Reyzelman, R.T. Crews, J.C. Moore, L. Moore, J.S. Mukker, S. Offutt, A. Tallis, W.B. Turner, D. Vayser, and C.J.I.w.j. Winters, Clinical effectiveness of an acellular dermal regenerative tissue matrix compared to standard wound management in healing diabetic foot ulcers: a prospective, randomised, multicentre study. 6 (2009) 196-208.

[77] C.M. Zelen, D.P. Orgill, T. Serena, R. Galiano, M.J. Carter, L.A. DiDomenico, J. Keller, J. Kaufman, and W.W.J.I.w.j. Li, A prospective, randomised, controlled, multicentre clinical trial examining healing rates, safety and cost to closure of an acellular reticular allogenic human dermis versus standard of care in the treatment of chronic diabetic foot ulcers. 14 (2017) 307-315.

[78] C.M. Zelen, D.P. Orgill, T.E. Serena, R.E. Galiano, M.J. Carter, L.A. DiDomenico, J. Keller, J.P. Kaufman, and W.W.J.I.w.j. Li, An aseptically processed, acellular, reticular, allogenic human dermis improves healing in diabetic foot ulcers: a prospective, randomised, controlled, multicentre follow‐up trial. 15 (2018) 731-739.

[79] H. Gu, X. Zhao, Y. Sun, Y. Ding, and R.J.S. Ouyang, Negative-pressure wound therapy compared with advanced moist wound therapy: A comparative study on healing efficacy in diabetic foot ulcers. 180 (2025) 109098.

[80] A.M. Tofigh, M.J.D.R. Tajik, and C. Practice, Comparing the standard surgical dressing with dehydrated amnion and platelet-derived growth factor dressings in the healing rate of diabetic foot ulcer: A randomized clinical trial. 185 (2022) 109775.

[81] D.G. Armstrong, R.D. Galiano, D.P. Orgill, P.M. Glat, M.J. Carter, L.A. Di Domenico, A.M. Reyzelman, and C.M.J.I.w.j. Zelen, Multi‐centre prospective randomised controlled clinical trial to evaluate a bioactive split thickness skin allograft vs standard of care in the treatment of diabetic foot ulcers. 19 (2022) 932-944.

[82] E.J. Lullove, B. Liden, P. McEneaney, A. Raphael, R. Klein, C. Winters, J.J.W.a.C.o.C.R. Lantis Ii, and Practice, Evaluating the effect of omega-3-rich fish skin in the treatment of chronic, nonresponsive diabetic foot ulcers: penultimate analysis of a multicenter, prospective, randomized controlled trial. 34 (2022) E34-E36.

[83] D.G. Armstrong, D.P. Orgill, R.D. Galiano, P.M. Glat, L.A. DiDomenico, M.J. Carter, and C.M.J.I.w.j. Zelen, A multi‐centre, single‐blinded randomised controlled clinical trial evaluating the effect of resorbable glass fibre matrix in the treatment of diabetic foot ulcers. 19 (2022) 791-801.

[84] D.G. Armstrong, D.P. Orgill, R.D. Galiano, P.M. Glat, J.P. Kaufman, M.J. Carter, L.A. DiDomenico, and C.M.J.I.w.j. Zelen, Use of a purified reconstituted bilayer matrix in the management of chronic diabetic foot ulcers improves patient outcomes vs standard of care: results of a prospective randomised controlled multi‐centre clinical trial. 19 (2022) 1197-1209.

FIGURE S1

ROB2 risk of bias assessment.

| **Intervention comparison** | **Outcome measure** | **Number** | **Effect size** | **I²** |
| --- | --- | --- | --- | --- |
| OXY vs SOC | Ulcer area reduction | 7 | SMD = 0.44 (95% CI 0.11 to 0.78) | 67.8% |
| DEV vs SOC | Ulcer area reduction | 7 | SMD = 1.52 (95% CI 0.61 to 2.42) | 94.5% |
| GFB vs SOC | Ulcer area reduction | 8 | SMD = 0.74 (95% CI 0.19 to 1.29) | 89.9% |
| PLA vs SOC | Ulcer area reduction | 5 | SMD = 1.25 (95% CI 0.03 to 2.47) | 94.5% |
| MSS vs SOC | Ulcer area reduction | 6 | SMD = 0.45 (95% CI 0.08 to 0.82) | 65.9% |
| TBA vs SOC | Ulcer area reduction | 3 | SMD = 0.13 (95% CI -0.26 to 0.52) | 17.4% |
| OXY vs SOC | Complete wound healing | 11 | OR = 2.52 (95% CI 1.57 to 4.04) | 32.5% |
| DEV vs SOC | Complete wound healing | 12 | OR = 1.79 (95% CI 1.36 to 2.37) | 38.2% |
| GFB vs SOC | Complete wound healing | 17 | OR = 1.41 (95% CI 1.17 to 1.71) | 0.0% |
| PLA vs SOC | Complete wound healing | 9 | OR = 1.77 (95% CI 1.32 to 2.38) | 0.0% |
| MSS vs SOC | Complete wound healing | 13 | OR = 2.08 (95% CI 1.65 to 2.62) | 0.0% |
| CAD vs SOC | Complete wound healing | 5 | OR = 1.56 (95% CI 0.90 to 2.69) | 0.0% |
| TBA vs SOC | Complete wound healing | 6 | OR = 1.58 (95% CI 1.16 to 2.14) | 0.0% |
| NND vs SOC | Complete wound healing | 2 | OR = 1.54 (95% CI 0.75 to 3.18) | 0.0% |
| OXY vs SOC | Healing Time | 3 | SMD = -0.53 (95% CI -1.09 to 0.03) | 67.5% |
| DEV vs SOC | Healing Time | 5 | SMD = -0.77 (95% CI -1.58 to 0.05) | 97.1% |
| GFB vs SOC | Healing Time | 9 | SMD = -1.17 (95% CI -1.83 to -0.52) | 90.3% |
| PLA vs SOC | Healing Time | 3 | SMD = -0.87 (95% CI -1.77 to 0.02) | 82.8% |
| MSS vs SOC | Healing Time | 6 | SMD = -0.89 (95% CI -1.38 to -0.41) | 76.9% |
| CAD vs SOC | Healing Time | 3 | SMD = -1.55 (95% CI -2.45 to -0.65) | 74.5% |
| OXY vs SOC | Adverse Event | 6 | OR = 0.86 (95% CI 0.56 to 1.33) | 0.0% |
| DEV vs SOC | Adverse Event | 4 | OR = 1.16 (95% CI 0.91 to 1.47) | 0.0% |
| GFB vs SOC | Adverse Event | 5 | OR = 1.07 (95% CI 0.75 to 1.53) | 0.0% |
| PLA vs SOC | Adverse Event | 7 | OR = 0.51 (95% CI 0.30 to 0.89) | 5.4% |
| MSS vs SOC | Adverse Event | 9 | OR = 0.77 (95% CI 0.52 to 1.14) | 0.0% |
| TBA vs SOC | Adverse Event | 4 | OR = 0.89 (95% CI 0.66 to 1.20) | 0.0% |
| OXY vs SOC | Amputation Rate | 5 | OR = 0.27 (95% CI 0.10 to 0.71) | 61.4% |
| DEV vs SOC | Amputation Rate | 5 | OR = 0.72 (95% CI 0.46 to 1.14) | 30.3% |
| GFB vs SOC | Amputation Rate | 4 | OR = 0.32 (95% CI 0.13 to 0.77) | 0.0% |

Table S4 Forest plots of the efficacy and safety outcomes of diabetic foot ulcer patients with different interventions.

Figure S2 Ulcer area reduction-OXY - Forest Plot

Figure S3 Ulcer area reduction-DEV - Forest Plot

Figure S4 Ulcer area reduction-GFB - Forest Plot

Figure S5 Ulcer area reduction-PLA - Forest Plot

Figure S6 Ulcer area reduction-MSS - Forest Plot

Figure S7 Ulcer area reduction-TBA - Forest Plot

Figure S8 Ulcer area reduction-NND - Forest Plot

Figure S9 Complete wound healing-OXY - Forest Plot

Figure S10 Complete wound healing-DEV - Forest Plot

Figure S11 Complete wound healing-GFB - Forest Plot

Figure S12 Complete wound healing-PLA - Forest Plot

Figure S13 Complete wound healing-MSS - Forest Plot

Figure S14 Complete wound healing-CAD - Forest Plot

Figure S15 Complete wound healing-TBA - Forest Plot

Figure S16 Complete wound healing-NND - Forest Plot

Figure S17 Healing Time-OXY - Forest Plot

Figure S18 Healing Time-DEV - Forest Plot

Figure S19 Healing Time-GFB - Forest Plot

Figure S20 Healing Time-PLA - Forest Plot

Figure S21 Healing Time-MSS - Forest Plot

Figure S22 Healing Time-CAD - Forest Plot

Figure S23 Adverse Event -OXY - Forest Plot

Figure S24 Adverse Event -DEV - Forest Plot

Figure S25 Adverse Event -GFB - Forest Plot

Figure S26 Adverse Event -PLA - Forest Plot

Figure S27 Adverse Event -MSS - Forest Plot

Figure S28 Adverse Event -TBA - Forest Plot

Figure S29 Amputation Rate -OXY - Forest Plot

Figure S30 Amputation Rate-DEV - Forest Plot

Figure S31 Amputation Rate - GFB - Forest Plot

Figure S32 Amputation Rate - MSS - Forest Plot

Figure S33 Amputation Rate - CAD - Forest Plot

Figure S34 Ulcer area reduction-OXY - Subgroup Analysis

Figure S35 Ulcer area reduction-DEV - Subgroup Analysis

Figure S36 Ulcer area reduction-GFB - Subgroup Analysis

Figure S37 Ulcer area reduction-PLA - Subgroup Analysis

Figure S38 Ulcer area reduction-MSS - Subgroup Analysis

Figure S39 Complete wound healing-OXY - Subgroup Analysis

Figure S40 Complete wound healing-DEV - Subgroup Analysis

Figure S41 Complete wound healing-GFB - Subgroup Analysis

Figure S42 Complete wound healing-PLA - Subgroup Analysis

Figure S43 Complete wound healing-MSS - Subgroup Analysis

Figure S44 Complete wound healing - CAD - Subgroup Analysis

Figure S45 Complete wound healing - TBA - Subgroup Analysis

Figure S46 Healing Time-DEV - Subgroup Analysis

Figure S47 Healing Time- GFB - Subgroup Analysis

Figure S48 Healing Time- MSS - Subgroup Analysis

Figure S49 Adverse Event -OXY - Subgroup Analysis

Figure S50 Adverse Event -DEV - Subgroup Analysis

Figure S51 Adverse Event -GFB- Subgroup Analysis

Figure S52 Adverse Event -PLA- Subgroup Analysis

Figure S53 Adverse Event -MSS- Subgroup Analysis

Figure S54 Adverse Event -TBA- Subgroup Analysis

Figure S55 Amputation Rate -OXY - Subgroup Analysis

Figure S56 Amputation Rate -DEV - Subgroup Analysis

Figure S57 Amputation Rate -GFB - Subgroup Analysis

Figure S58 Ulcer area reduction-OXY - Leave-One-Out Sensitivity

Figure S59 Ulcer area reduction- DEV - Leave-One-Out Sensitivity

Figure S60 Ulcer area reduction- GFB - Leave-One-Out Sensitivity

Figure S61 Ulcer area reduction- PLA - Leave-One-Out Sensitivity

Figure S62 Ulcer area reduction- MSS - Leave-One-Out Sensitivity

Figure S63 Ulcer area reduction- TBA - Leave-One-Out Sensitivity

Figure S64 Complete wound healing-OXY - Leave-One-Out Sensitivity

Figure S65 Complete wound healing-DEV- Leave-One-Out Sensitivity

Figure S66 Complete wound healing-GFB- Leave-One-Out Sensitivity

Figure S67 Complete wound healing-PLA- Leave-One-Out Sensitivity

Figure S68 Complete wound healing-MSS- Leave-One-Out Sensitivity

Figure S69 Complete wound healing-CAD- Leave-One-Out Sensitivity

Figure S70 Complete wound healing-TBA- Leave-One-Out Sensitivity

Figure S71 Healing Time- OXY - Leave-One-Out Sensitivity

Figure S72 Healing Time- DEV - Leave-One-Out Sensitivity

Figure S73 Healing Time- GFB - Leave-One-Out Sensitivity

Figure S74 Healing Time- PLA - Leave-One-Out Sensitivity

Figure S75 Healing Time- MSS - Leave-One-Out Sensitivity

Figure S76 Healing Time- CAD - Leave-One-Out Sensitivity

Figure S77 Adverse Event -OXY - Leave-One-Out Sensitivity

Figure S78 Adverse Event -DEV - Leave-One-Out Sensitivity

Figure S79 Adverse Event -GFB- Leave-One-Out Sensitivity

Figure S80 Adverse Event -PLA- Leave-One-Out Sensitivity

Figure S81 Adverse Event -MSS- Leave-One-Out Sensitivity

Figure S82 Adverse Event -TBA- Leave-One-Out Sensitivity

Figure S83 Amputation Rate -OXY - Leave-One-Out Sensitivity

Figure S84 Amputation Rate -DEV - Leave-One-Out Sensitivity

Figure S85 Amputation Rate -GFB - Leave-One-Out Sensitivity

Figure S86 Ulcer area reduction-OXY - Meta-Regression

Figure S87 Ulcer area reduction-DEV - Meta-Regression

Figure S88 Ulcer area reduction-GFB - Meta-Regression

Figure S89 Ulcer area reduction - PLA - Meta-Regression

Figure S90 Ulcer area reduction-MSS - Meta-Regression

Figure S91 Complete wound healing-OXY - Meta-Regression

Figure S92 Complete wound healing-DEV - Meta-Regression

Figure S93 Complete wound healing-GFB - Meta-Regression

Figure S94 Complete wound healing-PLA - Meta-Regression

Figure S95 Complete wound healing-MSS - Meta-Regression

Figure S96 Complete wound healing-CAD - Meta-Regression

Figure S97 Complete wound healing-TBA - Meta-Regression

Figure S98 Healing Time-DEV - Meta-Regression

Figure S99 Healing Time-GFB - Meta-Regression

Figure S100 Healing Time-MSS - Meta-Regression

Figure S101 Adverse Event -OXY - Meta-Regression

Figure S102 Adverse Event -DEV - Meta-Regression

Figure S103 Adverse Event -GFB - Meta-Regression

Figure S104 Adverse Event -PLA- Meta-Regression

Figure S105 Adverse Event -MSS - Meta-Regression

Figure S106 Adverse Event -TBA - Meta-Regression

Figure S107 Amputation Rate -OXY - Meta-Regression

Figure S108 Amputation Rate -DEV - Meta-Regression

Figure S109 Amputation Rate -GFB - Meta-Regression

| Leave-one-out analysis | **n** | Original P value | Minimum P value after omission | Maximum P value after omission |
| --- | --- | --- | --- | --- |
| Ulcer area reduction-DEV | 7 | <0.001 | <0.001 | 0.008 |
| Ulcer area reduction-GFB | 8 | 0.008 | 0.005 | 0.029 |
| Ulcer area reduction-MSS | 6 | 0.016 | 0.007 | 0.057 |
| Ulcer area reduction-OXY | 7 | 0.010 | 0.006 | 0.043 |
| Ulcer area reduction-PLA | 5 | 0.044 | 0.029 | 0.157 |
| Ulcer area reduction-TBA | 3 | 0.514 | 0.152 | 0.866 |
| Complete wound healing -CAD | 5 | 0.111 | 0.094 | 0.266 |
| Complete wound healing -DEV | 12 | <0.001 | <0.001 | <0.001 |
| Complete wound healing -GFB | 17 | <0.001 | <0.001 | 0.001 |
| Complete wound healing -MSS | 13 | <0.001 | <0.001 | <0.001 |
| Complete wound healing -OXY | 11 | <0.001 | <0.001 | <0.001 |
| Complete wound healing -PLA | 9 | <0.001 | <0.001 | 0.002 |
| Complete wound healing -TBA | 6 | 0.003 | 0.002 | 0.084 |
| Healing Time-CAD | 3 | <0.001 | <0.001 | 0.038 |
| Healing Time-DEV | 5 | 0.065 | 0.033 | 0.139 |
| Healing Time-GFB | 9 | <0.001 | <0.001 | 0.003 |
| Healing Time-MSS | 6 | <0.001 | <0.001 | 0.004 |
| Healing Time-OXY | 3 | 0.062 | <0.001 | 0.339 |
| Healing Time-PLA | 3 | 0.055 | <0.001 | 0.443 |
| Adverse Event -DEV | 4 | 0.227 | 0.170 | 0.826 |
| Adverse Event -GFB | 5 | 0.710 | 0.614 | 0.909 |
| Adverse Event -MSS | 9 | 0.189 | 0.140 | 0.435 |
| Adverse Event -OXY | 6 | 0.497 | 0.365 | 0.969 |
| Adverse Event -PLA | 7 | 0.017 | 0.011 | 0.049 |
| Adverse Event -TBA | 4 | 0.448 | 0.480 | 0.539 |
| Amputation Rate -DEV | 5 | 0.160 | 0.088 | 0.473 |
| Amputation Rate -GFB | 4 | 0.011 | 0.012 | 0.039 |
| Amputation Rate -OXY | 5 | 0.008 | <0.001 | 0.059 |

Table S5 Leave-one-out sensitivity analysis p-value summary

Figure S110 Complete wound healing-OXY - Funnel Plot

Figure S111 Complete wound healing-DEV - Funnel Plot

Figure S112 Complete wound healing-GFB - Funnel Plot

Figure S113 Complete wound healing-MSS - Funnel Plot

| Prob>chi2 | 0.6979 |
| --- | --- |
| Prob>chi2 | 0.1557 |
| Prob>chi2 | 0.2101 |

Table S6 For ulcer area reduction, complete wound healing, healing time, and adverse events, global inconsistency was evaluated

| Side | Direct |  | Indirect |  | Difference |  |  | tau |
| --- | --- | --- | --- | --- | --- | --- | --- | --- |
|  | Coef. | Std. Err. | Coef. | Std. Err. | Coef. | Std. Err. | P>z |  |
| DEV SOC* | -1.442738 | 0.2952605 | -0.33149 | 59.70731 | -1.111248 | 59.70802 | 0.985 | 0.6948316 |
| GFB MSS | 0.1302799 | 0.7617209 | -0.3458264 | 0.4427857 | 0.4761064 | 0.8803135 | 0.589 | 0.708834 |
| GFB PLA | 0.18 | 0.7315248 | 0.3132035 | 0.5228827 | -0.1332035 | 0.8991813 | 0.882 | 0.7144009 |
| GFB SOC* | -0.7166468 | 0.2735137 | -1.182125 | 1.083153 | 0.4654778 | 1.11952 | 0.678 | 0.7117562 |
| MSS SOC* | -0.4770498 | 0.3118462 | -1.384189 | 1.430225 | 0.9071397 | 1.465595 | 0.536 | 0.7080925 |
| NND SOC* | -0.2337419 | 0.8040989 | -2.830845 | 632.1792 | 2.597103 | 632.1798 | 0.997 | 0.6948309 |
| OXY SOC* | -0.4714534 | 0.2844184 | -2.885353 | 238.5297 | 2.413899 | 238.5299 | 0.992 | 0.694831 |
| PLA SOC* | -0.954691 | 0.3785295 | -1.778829 | 1.378784 | 0.8241377 | 1.42855 | 0.564 | 0.7066498 |
| SOC TBA* | 0.2828893 | 0.4717622 | 2.871122 | 364.2663 | -2.588233 | 364.2666 | 0.994 | 0.6948304 |

Table S7 Local inconsistency assessment for ulcer area reduction

| Side | Direct |  | Indirect |  | Difference |  |  | tau |
| --- | --- | --- | --- | --- | --- | --- | --- | --- |
|  | Coef. | Std. Err. | Coef. | Std. Err. | Coef. | Std. Err. | P>z |  |
| CAD SOC* | -1.539327 | 0.585361 | -0.6547339 | 84.99105 | -0.8845929 | 84.99306 | 0.992 | 0.6125963 |
| DEV OXY | -1.098612 | 0.7656776 | 0.356671 | 0.3710108 | -1.455283 | 0.8508297 | 0.087 | 0.6016807 |
| DEV SOC | -1.045684 | 0.2312352 | -2.500969 | 0.8190429 | 1.455285 | 0.8508264 | 0.087 | 0.601679 |
| GFB MSS | -0.1281549 | 0.8444513 | 0.3484396 | 0.3324884 | -0.4765945 | 0.9072772 | 0.599 | 0.6201667 |
| GFB OXY | -0.9757339 | 0.9462001 | 0.2054085 | 0.3711085 | -1.181142 | 1.017775 | 0.246 | 0.6152851 |
| GFB SOC* | -1.128299 | 0.2266672 | -2.842646 | 1.181272 | 1.714347 | 1.206003 | 0.155 | 0.6140027 |
| MSS PLA | 2.056745 | 1.136249 | -0.1193142 | 0.4066212 | 2.17606 | 1.192233 | 0.068 | 0.6057046 |
| MSS SOC* | -1.520984 | 0.2318007 | -0.3146373 | 1.288235 | -1.206347 | 1.311757 | 0.358 | 0.6177312 |
| NND SOC* | -1.354459 | 0.780228 | -3.071177 | 1179.151 | 1.716718 | 1179.151 | 0.999 | 0.6125961 |
| OXY SOC | -1.452359 | 0.2931957 | 0.1342113 | 0.7195236 | -1.58657 | 0.7765895 | 0.051 | 0.5935015 |
| PLA SOC* | -1.560116 | 0.3273324 | -3.575097 | 1.623321 | 2.014981 | 1.613304 | 0.212 | 0.6165972 |
| SOC TBA* | 0.7406485 | 0.3314777 | 3.030969 | 548.5193 | -2.29032 | 548.5194 | 0.997 | 0.6125964 |

Table S8 Local inconsistency assessment for complete wound healing

| Side | Direct |  | Indirect |  | Difference |  |  | tau |
| --- | --- | --- | --- | --- | --- | --- | --- | --- |
|  | Coef. | Std. Err. | Coef. | Std. Err. | Coef. | Std. Err. | P>z |  |
| CAD SOC* | 1.510989 | 0.4944634 | 0.4725765 | 64.71979 | 1.038413 | 64.72169 | 0.987 | 0.7487367 |
| DEV SOC* | 0.7338903 | 0.3506212 | 3.057746 | 282.7567 | -2.323856 | 282.7569 | 0.993 | 0.7487368 |
| GFB OXY | -0.2956246 | 0.812923 | 0.7863026 | 0.6153146 | -1.081927 | 1.019635 | 0.289 | 0.7483536 |
| GFB SOC* | 1.136914 | 0.2817167 | 1.286356 | 1.845098 | -0.1494418 | 1.866419 | 0.936 | 0.7698418 |
| MSS PLA | -0.9768683 | 0.7796357 | 0.4540445 | 0.6780719 | -1.430913 | 1.032318 | 0.166 | 0.7356974 |
| MSS SOC* | 0.895373 | 0.3294044 | -1.284087 | 1.824332 | 2.17946 | 1.854258 | 0.24 | 0.7463691 |
| OXY SOC* | 0.5150324 | 0.4408953 | 3.265531 | 1.458302 | -2.750498 | 1.523559 | 0.071 | 0.709421 |
| PLA SOC* | 0.8314291 | 0.4795876 | 2.599528 | 1.545143 | -1.768098 | 1.616412 | 0.274 | 0.7475636 |

Table S9 Local inconsistency assessment for healing time

| Loop | IF | seIF | z_value | p_value | CI_95 | Loop_Heterog |
| --- | --- | --- | --- | --- | --- | --- |
| tau2 |  |  |  |  |  |  |
| CC-CC-E2-G | 0.401 | 0.778 | 0.516 | 0.606 | (0.00,1.93) | 0 |
| 0.371 |  |  |  |  |  |  |
| CC-F-G | 0.028 | 1.054 | 0.027 | 0.979 | (0.00,2.09) | 0 |
| 0.632 |  |  |  |  |  |  |

Table S10 Ring inconsistency analysis of ulcer area reduction

| Loop | IF | seIF | z_value | p_value | CI_95 | Loop_Heterog |
| --- | --- | --- | --- | --- | --- | --- |
| rog_tau2 |  |  |  |  |  |  |
| CC-MET-G-H | 2.497 | 1.296 | 1.927 | 0.054 | (0.00,5.04) |  |
| 0.209 |  |  |  |  |  |  |
| CC-F-H | 1.649 | 1.212 | 1.36 | 0.174 | (0.00,4.03) |  |
| 0.919 |  |  |  |  |  |  |
| CC-E2-F-H | 1.322 | 1.027 | 1.287 | 0.198 | (0.00,3.34) |  |
| 0.261 |  |  |  |  |  |  |
| CC-E2-CC-MET-H | 0.627 | 0.663 | 0.947 | 0.344 | (0.00,1.93) |  |
| 0.048 |  |  |  |  |  |  |

Table S11 Ring inconsistency analysis of complete wound healing

| Loop | IF | seIF | z_value | p_value | CI_95 | Loop_Heterog |
| --- | --- | --- | --- | --- | --- | --- |
| rog_tau2 |  |  |  |  |  |  |
| CC-E2-CC-NAC-G | 1.104 | 0.956 | 1.155 | 0.248 | (0.00,2.98) |  |
| 0.529 |  |  |  |  |  |  |
| CC-MET-F-G | 1.09 | 0.776 | 1.404 | 0.16 | (0.00,2.61) |  |
| 0.284 |  |  |  |  |  |  |

Table S12 Ring inconsistency analysis of healing time

Figure S114 SUCRA rank plot for ulcer area reduction outcome in adult patients with diabetes mellitus and below-ankle diabetic foot ulcers

| Treatment | SUCRA | PrBest | MeanRank |
| --- | --- | --- | --- |
| DEV | 95.3 | 74.7 | 1.3 |
| PLA | 76.4 | 14.7 | 2.7 |
| GFB | 61.6 | 1.8 | 3.7 |
| MSS | 46.3 | 0.5 | 4.8 |
| OXY | 42.8 | 0.4 | 5 |
| NND | 34.6 | 6.7 | 5.6 |
| TBA | 32.3 | 1.2 | 5.7 |
| SOC | 10.7 | 0 | 7.2 |

Table S13 SUCRA ranking table for ulcer area reduction outcome in adult patients with diabetes mellitus and below-ankle diabetic foot ulcers

Figure S115 SUCRA rank plot for complete wound healing outcome in adult patients with diabetes mellitus and below-ankle diabetic foot ulcers

| Treatment | SUCRA | PrBest | MeanRank |
| --- | --- | --- | --- |
| PLA | 77.9 | 25.6 | 2.8 |
| MSS | 72.1 | 11.6 | 3.2 |
| CAD | 69.7 | 30.8 | 3.4 |
| NND | 59.1 | 26.8 | 4.3 |
| OXY | 53.2 | 2.8 | 4.7 |
| GFB | 49.2 | 1.3 | 5.1 |
| DEV | 45.5 | 0.9 | 5.4 |
| TBA | 22.5 | 0.1 | 7.2 |
| SOC | 0.8 | 0 | 8.9 |

Table S14 SUCRA ranking table for complete wound healing outcome in adult patients with diabetes mellitus and below-ankle diabetic foot ulcers

Figure S116 SUCRA rank plot for healing time outcome in adult patients with diabetes mellitus and below-ankle diabetic foot ulcers

| Treatment | SUCRA | PrBest | MeanRank |
| --- | --- | --- | --- |
| CAD | 86 | 59.9 | 1.8 |
| GFB | 70.4 | 15 | 2.8 |
| PLA | 59.4 | 13.9 | 3.4 |
| MSS | 48.5 | 3.7 | 4.1 |
| OXY | 42.6 | 5.1 | 4.4 |
| DEV | 41.8 | 2.4 | 4.5 |
| SOC | 1.4 | 0 | 6.9 |

Table S15 SUCRA ranking table for healing time outcome in adult patients with diabetes mellitus and below-ankle diabetic foot ulcers

Figure S117 SUCRA rank plot for adverse events outcome in adult patients with diabetes mellitus and below-ankle diabetic foot ulcers

| Treatment | SUCRA | PrBest | MeanRank |
| --- | --- | --- | --- |
| PLA | 98.7 | 93.8 | 1.1 |
| MSS | 73.7 | 3.9 | 2.6 |
| TBA | 66.8 | 2.1 | 3 |
| OXY | 48 | 0.2 | 4.1 |
| SOC | 35 | 0 | 4.9 |
| GFB | 23 | 0 | 5.6 |
| DEV | 4.7 | 0 | 6.7 |

Table S16 SUCRA ranking table for adverse event outcome in adult patients with diabetes mellitus and below-ankle diabetic foot ulcers

Figure S118 SUCRA rank plot for amputation rate outcome in adult patients with diabetes mellitus and below-ankle diabetic foot ulcers

| Treatment | SUCRA | PrBest | MeanRank |
| --- | --- | --- | --- |
| OXY | 84.8 | 45.3 | 1.8 |
| GFB | 72.4 | 21.8 | 2.4 |
| CAD | 49.4 | 20.5 | 3.5 |
| DEV | 39 | 0.3 | 4.1 |
| MSS | 35.9 | 12.1 | 4.2 |
| SOC | 18.6 | 0 | 5.1 |

Table S17 SUCRA ranking table for amputation rate outcome in adult patients with diabetes mellitus and below-ankle diabetic foot ulcers

|  |  | Coefficient | Std. err. | z | P>z | [95%  conf. | interval] |
| --- | --- | --- | --- | --- | --- | --- | --- |
| _y_B |  |  |  |  |  |  |  |
|  | week | -0.0676797 | 0.1172138 | -0.58 | 0.564 | -0.2974145 | 0.1620552 |
|  | _cons | -0.3851403 | 1.009125 | -0.38 | 0.703 | -2.36299 | 1.592709 |
|  |  |  |  |  |  |  |  |
| _y_C |  |  |  |  |  |  |  |
|  | week | -0.1376853 | 0.1426349 | -0.97 | 0.334 | -0.4172446 | 0.141874 |
|  | _cons | 0.0062452 | 1.360147 | 0 | 0.996 | -2.659593 | 2.672083 |
|  |  |  |  |  |  |  |  |
| _y_D |  |  |  |  |  |  |  |
|  | _cons | -1.619753 | 0.9466774 | -1.71 | 0.087 | -3.475206 | 0.2357011 |
|  |  |  |  |  |  |  |  |
| _y_E |  |  |  |  |  |  |  |
|  | week | -0.1969413 | 0.1238918 | -1.59 | 0.112 | -0.4397647 | 0.0458822 |
|  | _cons | 0.4664611 | 1.011651 | 0.46 | 0.645 | -1.516338 | 2.44926 |
|  |  |  |  |  |  |  |  |
| _y_F |  |  |  |  |  |  |  |
|  | week | -0.1453896 | 0.1331457 | -1.09 | 0.275 | -0.4063503 | 0.1155712 |
|  | _cons | 0.6261702 | 1.194867 | 0.52 | 0.6 | -1.715726 | 2.968066 |
|  |  |  |  |  |  |  |  |
| _y_G |  |  |  |  |  |  |  |
|  | week | -0.1347162 | 0.0988238 | -1.36 | 0.173 | -0.3284074 | 0.0589749 |
|  | _cons | -0.5063384 | 0.7579305 | -0.67 | 0.504 | -1.991855 | 0.979178 |
|  |  |  |  |  |  |  |  |
| _y_H |  |  |  |  |  |  |  |
|  | week | -0.262072 | 0.268573 | -0.98 | 0.329 | -0.7884653 | 0.2643214 |
|  | _cons | 1.531361 | 3.547998 | 0.43 | 0.666 | -5.422587 | 8.485309 |

Table S18 Meta-regression for ulcer area reduction outcome in adult patients with diabetes mellitus and below-ankle diabetic foot ulcers

|  |  | Coefficient | Std. err. | z | P>z | [95%  conf. | interval] |
| --- | --- | --- | --- | --- | --- | --- | --- |
| _y_B |  |  |  |  |  |  |  |
|  | week | -0.0965274 | 0.3188081 | -0.3 | 0.762 | -0.7213797 | 0.528325 |
|  | _cons | 0.6774371 | 2.855215 | 0.24 | 0.812 | -4.918681 | 6.273555 |
|  |  |  |  |  |  |  |  |
| _y_C |  |  |  |  |  |  |  |
|  | week | -0.0697369 | 0.3190152 | -0.22 | 0.827 | -0.6949953 | 0.5555215 |
|  | _cons | 0.3661376 | 2.852368 | 0.13 | 0.898 | -5.224401 | 5.956676 |
|  |  |  |  |  |  |  |  |
| _y_D |  |  |  |  |  |  |  |
|  | week | -0.0610645 | 0.3323016 | -0.18 | 0.854 | -0.7123637 | 0.5902347 |
|  | _cons | 0.5487264 | 3.043284 | 0.18 | 0.857 | -5.416001 | 6.513454 |
|  |  |  |  |  |  |  |  |
| _y_E |  |  |  |  |  |  |  |
|  | week | -0.2034929 | 0.4930477 | -0.41 | 0.68 | -1.169849 | 0.7628627 |
|  | _cons | 0.9088371 | 3.409711 | 0.27 | 0.79 | -5.774074 | 7.591748 |
|  |  |  |  |  |  |  |  |
| _y_F |  |  |  |  |  |  |  |
|  | week | -0.0807987 | 0.3249165 | -0.25 | 0.804 | -0.7176233 | 0.5560259 |
|  | _cons | 0.4920275 | 2.896577 | 0.17 | 0.865 | -5.18516 | 6.169215 |
|  |  |  |  |  |  |  |  |
| _y_G |  |  |  |  |  |  |  |
|  | week | -0.180919 | 0.3330825 | -0.54 | 0.587 | -0.8337487 | 0.4719106 |
|  | _cons | 1.867183 | 3.017586 | 0.62 | 0.536 | -4.047177 | 7.781543 |
|  |  |  |  |  |  |  |  |
| _y_H |  |  |  |  |  |  |  |
|  | week | -0.0297524 | 0.3147757 | -0.09 | 0.925 | -0.6467014 | 0.5871967 |
|  | _cons | -1.280554 | 2.782057 | -0.46 | 0.645 | -6.733286 | 4.172177 |
|  |  |  |  |  |  |  |  |
| _y_I |  |  |  |  |  |  |  |
|  | week | 0.1443814 | 0.3565775 | 0.4 | 0.686 | -0.5544976 | 0.8432604 |
|  | _cons | -2.889136 | 3.599613 | -0.8 | 0.422 | -9.944247 | 4.165976 |

Table S19 Meta-regression for complete wound healing outcome in adult patients with diabetes mellitus and below-ankle diabetic foot ulcers

|  |  | Coefficient | Std. err. | z | P>z | [95%  conf. | interval] |
| --- | --- | --- | --- | --- | --- | --- | --- |
| _y_B |  |  |  |  |  |  |  |
|  | day | 0.1678723 | 0.1559414 | 1.08 | 0.282 | -0.1377673 | 0.4735119 |
|  | _cons | -6.220479 | 6.683857 | -0.93 | 0.352 | -19.3206 | 6.87964 |
|  |  |  |  |  |  |  |  |
| _y_C |  |  |  |  |  |  |  |
|  | day | 0.1706659 | 0.1554682 | 1.1 | 0.272 | -0.1340463 | 0.4753781 |
|  | _cons | -6.801619 | 6.597447 | -1.03 | 0.303 | -19.73238 | 6.12914 |
|  |  |  |  |  |  |  |  |
| _y_D |  |  |  |  |  |  |  |
|  | day | 0.1683649 | 0.1564948 | 1.08 | 0.282 | -0.1383593 | 0.4750891 |
|  | _cons | -6.381779 | 6.682041 | -0.96 | 0.34 | -19.47834 | 6.714781 |
|  |  |  |  |  |  |  |  |
| _y_E |  |  |  |  |  |  |  |
|  | day | 0.1692125 | 0.154667 | 1.09 | 0.274 | -0.1339293 | 0.4723542 |
|  | _cons | -6.237809 | 6.570369 | -0.95 | 0.342 | -19.11549 | 6.639878 |
|  |  |  |  |  |  |  |  |
| _y_F |  |  |  |  |  |  |  |
|  | day | 0.1063111 | 0.163247 | 0.65 | 0.515 | -0.2136471 | 0.4262693 |
|  | _cons | -3.815354 | 6.938678 | -0.55 | 0.582 | -17.41491 | 9.784205 |
|  |  |  |  |  |  |  |  |
| _y_G |  |  |  |  |  |  |  |
|  | day | 0.1707578 | 0.1545947 | 1.1 | 0.269 | -0.1322422 | 0.4737578 |
|  | _cons | -5.670316 | 6.529249 | -0.87 | 0.385 | -18.46741 | 7.126777 |

Table S20 Meta-regression for healing time outcome in adult patients with diabetes mellitus and below-ankle diabetic foot ulcers

|  |  | Coefficient | Std. err. | z | P>z | [95%  conf. | interval] |
| --- | --- | --- | --- | --- | --- | --- | --- |
| _y_B |  |  |  |  |  |  |  |
|  | week | -0.1407403 | 0.0792463 | -1.78 | 0.076 | -0.2960602 | 0.0145795 |
|  | _cons | 1.796455 | 1.173041 | 1.53 | 0.126 | -0.5026628 | 4.095573 |
|  |  |  |  |  |  |  |  |
| _y_C |  |  |  |  |  |  |  |
|  | week | -0.2210148 | 0.1364551 | -1.62 | 0.105 | -0.488462 | 0.0464323 |
|  | _cons | 2.107373 | 1.720566 | 1.22 | 0.221 | -1.264874 | 5.479619 |
|  |  |  |  |  |  |  |  |
| _y_D |  |  |  |  |  |  |  |
|  | week | -0.2106952 | 0.1225852 | -1.72 | 0.086 | -0.4509577 | 0.0295673 |
|  | _cons | 2.184426 | 1.435341 | 1.52 | 0.128 | -0.6287917 | 4.997643 |
|  |  |  |  |  |  |  |  |
| _y_E |  |  |  |  |  |  |  |
|  | week | -0.0829841 | 0.1182962 | -0.7 | 0.483 | -0.3148405 | 0.1488722 |
|  | _cons | -0.0683109 | 1.431348 | -0.05 | 0.962 | -2.873702 | 2.73708 |
|  |  |  |  |  |  |  |  |
| _y_F |  |  |  |  |  |  |  |
|  | week | -0.1095991 | 0.0693735 | -1.58 | 0.114 | -0.2455687 | 0.0263705 |
|  | _cons | 1.186718 | 0.9862469 | 1.2 | 0.229 | -0.7462906 | 3.119726 |
|  |  |  |  |  |  |  |  |
| _y_G |  |  |  |  |  |  |  |
|  | week | -0.0659354 | 0.1232742 | -0.53 | 0.593 | -0.3075485 | 0.1756776 |
|  | _cons | 0.2573033 | 1.762547 | 0.15 | 0.884 | -3.197225 | 3.711832 |

Table S21 Meta-regression for adverse events outcome in adult patients with diabetes mellitus and below-ankle diabetic foot ulcers

|  |  | Coefficient | Std. err. | z | P>z | [95%  conf. | interval] |
| --- | --- | --- | --- | --- | --- | --- | --- |
| _y_B |  |  |  |  |  |  |  |
|  | week | 0.1108076 | 37.87533 | 0 | 0.998 | -74.12347 | 74.34509 |
|  | _cons | -1.721446 | 227.2962 | -0.01 | 0.994 | -447.2138 | 443.7709 |
|  |  |  |  |  |  |  |  |
| _y_C |  |  |  |  |  |  |  |
|  | week | 0.1462195 | 37.87739 | 0 | 0.997 | -74.09211 | 74.38455 |
|  | _cons | -2.308154 | 227.3107 | -0.01 | 0.992 | -447.8289 | 443.2126 |
|  |  |  |  |  |  |  |  |
| _y_D |  |  |  |  |  |  |  |
|  | _cons | 0.3094768 | 227.2494 | 0 | 0.999 | -445.0911 | 445.7101 |
|  |  |  |  |  |  |  |  |
| _y_E |  |  |  |  |  |  |  |
|  | week | -0.065901 | 37.8749 | 0 | 0.999 | -74.29934 | 74.16754 |
|  | _cons | -0.6551142 | 227.2704 | 0 | 0.998 | -446.0968 | 444.7866 |
|  |  |  |  |  |  |  |  |
| _y_F |  |  |  |  |  |  |  |
|  | week | -0.051733 | 37.87444 | 0 | 0.999 | -74.28427 | 74.1808 |
|  | _cons | 1.121328 | 227.2631 | 0 | 0.996 | -444.3062 | 446.5489 |

Table S22 Meta-regression for amputation rate outcome in adult patients with diabetes mellitus and below-ankle diabetic foot ulcers

Figure S119 Comparison-adjusted funnel plot for ulcer area reduction outcome in adult patients with diabetes mellitus and below-ankle diabetic foot ulcers

Figure S120 Comparison-adjusted funnel plot for complete wound healing outcome in adult patients with diabetes mellitus and below-ankle diabetic foot ulcers

Figure S121 Comparison-adjusted funnel plot for healing time outcome in adult patients with diabetes mellitus and below-ankle diabetic foot ulcers

Figure S122 Comparison-adjusted funnel plot for adverse events outcome in adult patients with diabetes mellitus and below-ankle diabetic foot ulcers

Figure S123 Comparison-adjusted funnel plot for amputation rate outcome in adult patients with diabetes mellitus and below-ankle diabetic foot ul

| Comparison | Number of studies | Within-study bias | Reporting bias | Indirectness | Imprecision | Heterogeneity | Incoherence | Confidence rating |
| --- | --- | --- | --- | --- | --- | --- | --- | --- |
| DEV:SOC | 7 | Major concerns | Low risk | No concerns | No concerns | No concerns | Some concerns | Very low |
| GFB:MSS | 1 | Some concerns | Low risk | No concerns | Some concerns | Some concerns | No concerns | Very low |
| GFB:PLA | 1 | Major concerns | Low risk | No concerns | No concerns | No concerns | No concerns | Low |
| GFB:SOC | 8 | Some concerns | Low risk | No concerns | No concerns | Some concerns | No concerns | Low |
| MSS:SOC | 6 | No concerns | Low risk | No concerns | No concerns | Some concerns | No concerns | Moderate |
| NND:SOC | 1 | Major concerns | Low risk | No concerns | No concerns | No concerns | Some concerns | Very low |
| OXY:SOC | 7 | Some concerns | Low risk | No concerns | Some concerns | No concerns | Some concerns | Very low |
| PLA:SOC | 5 | Major concerns | Low risk | No concerns | No concerns | No concerns | No concerns | Low |
| SOC:TBA | 3 | Some concerns | Low risk | No concerns | No concerns | No concerns | Some concerns | Low |
| DEV:GFB | 0 | Major concerns | Low risk | No concerns | No concerns | Some concerns | Some concerns | Very low |
| DEV:MSS | 0 | No concerns | Low risk | No concerns | No concerns | No concerns | Some concerns | Moderate |
| DEV:NND | 0 | Major concerns | Low risk | No concerns | No concerns | No concerns | Some concerns | Very low |
| DEV:OXY | 0 | Some concerns | Low risk | No concerns | No concerns | No concerns | Some concerns | Low |
| DEV:PLA | 0 | Major concerns | Low risk | No concerns | No concerns | No concerns | Some concerns | Very low |
| DEV:TBA | 0 | Some concerns | Low risk | No concerns | No concerns | No concerns | Some concerns | Low |
| GFB:NND | 0 | Major concerns | Low risk | No concerns | No concerns | No concerns | Some concerns | Very low |
| GFB:OXY | 0 | Some concerns | Low risk | No concerns | No concerns | No concerns | Some concerns | Low |
| GFB:TBA | 0 | Some concerns | Low risk | No concerns | No concerns | Some concerns | Some concerns | Very low |
| MSS:NND | 0 | Major concerns | Low risk | No concerns | Major concerns | No concerns | Some concerns | Very low |
| MSS:OXY | 0 | No concerns | Low risk | No concerns | Some concerns | No concerns | Major concerns | Very low |
| MSS:PLA | 0 | Major concerns | Low risk | No concerns | No concerns | No concerns | Some concerns | Very low |
| MSS:TBA | 0 | Some concerns | Low risk | No concerns | No concerns | No concerns | Some concerns | Low |
| NND:OXY | 0 | Major concerns | Low risk | No concerns | Major concerns | No concerns | Some concerns | Very low |
| NND:PLA | 0 | Major concerns | Low risk | No concerns | Some concerns | No concerns | Some concerns | Very low |
| NND:TBA | 0 | Major concerns | Low risk | No concerns | No concerns | No concerns | Some concerns | Very low |
| OXY:PLA | 0 | Major concerns | Low risk | No concerns | No concerns | No concerns | Some concerns | Very low |
| OXY:TBA | 0 | Some concerns | Low risk | No concerns | No concerns | No concerns | Some concerns | Low |
| PLA:TBA | 0 | Some concerns | Low risk | No concerns | No concerns | No concerns | Some concerns | Low |

Table S23 GRADE certainty of evidence for ulcer area reduction outcome in adult patients with diabetes mellitus and below-ankle diabetic foot ulcers

| Comparison | Number of studies | Within-study bias | Reporting bias | Indirectness | Imprecision | Heterogeneity | Incoherence | Confidence rating |
| --- | --- | --- | --- | --- | --- | --- | --- | --- |
| CAD:SOC | 5 | Some concerns | Low risk | No concerns | No concerns | Some concerns | Some concerns | Very low |
| DEV:OXY | 1 | No concerns | Low risk | No concerns | Some concerns | No concerns | No concerns | Moderate |
| DEV:SOC | 12 | No concerns | Low risk | No concerns | No concerns | Some concerns | No concerns | Moderate |
| GFB:MSS | 1 | No concerns | Low risk | No concerns | Some concerns | No concerns | No concerns | Moderate |
| GFB:OXY | 1 | No concerns | Low risk | No concerns | Some concerns | No concerns | No concerns | Moderate |
| GFB:SOC | 17 | No concerns | Low risk | No concerns | No concerns | No concerns | No concerns | High |
| MSS:PLA | 1 | No concerns | Low risk | No concerns | Some concerns | No concerns | Some concerns | Low |
| MSS:SOC | 13 | No concerns | Low risk | No concerns | No concerns | No concerns | No concerns | High |
| NND:SOC | 2 | Major concerns | Low risk | No concerns | Some concerns | No concerns | No concerns | Very low |
| OXY:SOC | 11 | No concerns | Low risk | No concerns | No concerns | No concerns | Some concerns | Moderate |
| PLA:SOC | 9 | No concerns | Low risk | No concerns | Some concerns | No concerns | No concerns | Moderate |
| SOC:TBA | 6 | No concerns | Low risk | No concerns | No concerns | Some concerns | Some concerns | Low |
| CAD:DEV | 0 | No concerns | Low risk | No concerns | No concerns | No concerns | Some concerns | Moderate |
| CAD:GFB | 0 | No concerns | Low risk | No concerns | No concerns | No concerns | Some concerns | Moderate |
| CAD:MSS | 0 | Some concerns | Low risk | No concerns | No concerns | No concerns | Some concerns | Low |
| CAD:NND | 0 | Major concerns | Low risk | No concerns | No concerns | No concerns | No concerns | Low |
| CAD:OXY | 0 | Some concerns | Low risk | No concerns | No concerns | No concerns | Some concerns | Low |
| CAD:PLA | 0 | No concerns | Low risk | No concerns | No concerns | No concerns | Some concerns | Moderate |
| CAD:TBA | 0 | Some concerns | Low risk | No concerns | No concerns | No concerns | No concerns | Moderate |
| DEV:GFB | 0 | No concerns | Low risk | No concerns | No concerns | No concerns | Some concerns | Moderate |
| DEV:MSS | 0 | No concerns | Low risk | No concerns | No concerns | No concerns | Major concerns | Low |
| DEV:NND | 0 | Major concerns | Low risk | No concerns | No concerns | No concerns | Some concerns | Very low |
| DEV:PLA | 0 | No concerns | Low risk | No concerns | No concerns | No concerns | No concerns | High |
| DEV:TBA | 0 | No concerns | Low risk | No concerns | No concerns | No concerns | Some concerns | Moderate |
| GFB:NND | 0 | Major concerns | Low risk | No concerns | No concerns | No concerns | No concerns | Low |
| GFB:PLA | 0 | No concerns | Low risk | No concerns | No concerns | No concerns | Some concerns | Moderate |
| GFB:TBA | 0 | No concerns | Low risk | No concerns | No concerns | No concerns | Some concerns | Moderate |
| MSS:NND | 0 | Major concerns | Low risk | No concerns | No concerns | No concerns | No concerns | Low |
| MSS:OXY | 0 | No concerns | Low risk | No concerns | No concerns | No concerns | Major concerns | Low |
| MSS:TBA | 0 | No concerns | Low risk | No concerns | No concerns | No concerns | No concerns | High |
| NND:OXY | 0 | Major concerns | Low risk | No concerns | No concerns | No concerns | Some concerns | Very low |
| NND:PLA | 0 | Major concerns | Low risk | No concerns | No concerns | No concerns | Some concerns | Very low |
| NND:TBA | 0 | Major concerns | Low risk | No concerns | No concerns | No concerns | Some concerns | Very low |
| OXY:PLA | 0 | No concerns | Low risk | No concerns | No concerns | No concerns | No concerns | High |
| OXY:TBA | 0 | No concerns | Low risk | No concerns | No concerns | No concerns | No concerns | High |
| PLA:TBA | 0 | No concerns | Low risk | No concerns | Some concerns | No concerns | Some concerns | Low |

Table S24 GRADE certainty of evidence for complete wound healing outcome in adult patients with diabetes mellitus and below-ankle diabetic foot ulcers

| Comparison | Number of studies | Within-study bias | Reporting bias | Indirectness | Imprecision | Heterogeneity | Incoherence | Confidence rating |
| --- | --- | --- | --- | --- | --- | --- | --- | --- |
| CAD:SOC | 3 | No concerns | Low risk | No concerns | No concerns | No concerns | Some concerns | Moderate |
| DEV:SOC | 5 | No concerns | Low risk | No concerns | No concerns | Some concerns | Some concerns | Low |
| GFB:OXY | 1 | No concerns | Low risk | No concerns | Some concerns | No concerns | No concerns | Moderate |
| GFB:SOC | 9 | No concerns | Low risk | No concerns | No concerns | No concerns | No concerns | High |
| MSS:PLA | 1 | Major concerns | Low risk | No concerns | Some concerns | No concerns | No concerns | Very low |
| MSS:SOC | 6 | No concerns | Low risk | No concerns | No concerns | Some concerns | No concerns | Moderate |
| OXY:SOC | 3 | No concerns | Low risk | No concerns | Some concerns | No concerns | Some concerns | Low |
| PLA:SOC | 3 | Major concerns | Low risk | No concerns | No concerns | Some concerns | No concerns | Very low |
| CAD:DEV | 0 | No concerns | Low risk | No concerns | Some concerns | No concerns | Some concerns | Low |
| CAD:GFB | 0 | No concerns | Low risk | No concerns | Some concerns | No concerns | Some concerns | Low |
| CAD:MSS | 0 | No concerns | Low risk | No concerns | Some concerns | No concerns | Some concerns | Low |
| CAD:OXY | 0 | No concerns | Low risk | No concerns | Some concerns | No concerns | Major concerns | Very low |
| CAD:PLA | 0 | No concerns | Low risk | No concerns | Some concerns | No concerns | Some concerns | Low |
| DEV:GFB | 0 | No concerns | Low risk | No concerns | Some concerns | No concerns | Major concerns | Very low |
| DEV:MSS | 0 | No concerns | Low risk | No concerns | Some concerns | No concerns | Some concerns | Low |
| DEV:OXY | 0 | No concerns | Low risk | No concerns | Some concerns | No concerns | Some concerns | Low |
| DEV:PLA | 0 | No concerns | Low risk | No concerns | Some concerns | No concerns | Major concerns | Very low |
| GFB:MSS | 0 | No concerns | Low risk | No concerns | Some concerns | No concerns | Some concerns | Low |
| GFB:PLA | 0 | No concerns | Low risk | No concerns | Some concerns | No concerns | Some concerns | Low |
| MSS:OXY | 0 | No concerns | Low risk | No concerns | Some concerns | No concerns | Some concerns | Low |
| OXY:PLA | 0 | No concerns | Low risk | No concerns | Some concerns | No concerns | Some concerns | Low |

Table S25 GRADE certainty of evidence for healing time outcome in adult patients with diabetes mellitus and below-ankle diabetic foot ulcers

| Comparison | Number of studies | Within-study bias | Reporting bias | Indirectness | Imprecision | Heterogeneity | Incoherence | Confidence rating |
| --- | --- | --- | --- | --- | --- | --- | --- | --- |
| DEV:OXY | 1 | Some concerns | Low risk | No concerns | No concerns | No concerns | Some concerns | Low |
| DEV:SOC | 4 | No concerns | Low risk | No concerns | No concerns | No concerns | Some concerns | Moderate |
| GFB:SOC | 5 | No concerns | Low risk | No concerns | Some concerns | No concerns | No concerns | Moderate |
| MSS:PLA | 1 | No concerns | Low risk | No concerns | No concerns | No concerns | No concerns | High |
| MSS:SOC | 9 | No concerns | Low risk | No concerns | No concerns | No concerns | No concerns | High |
| OXY:SOC | 6 | Some concerns | Low risk | No concerns | Some concerns | No concerns | No concerns | Low |
| PLA:SOC | 7 | No concerns | Low risk | No concerns | No concerns | No concerns | No concerns | High |
| SOC:TBA | 4 | Some concerns | Low risk | No concerns | No concerns | No concerns | No concerns | Moderate |
| DEV:GFB | 0 | No concerns | Low risk | No concerns | Some concerns | No concerns | No concerns | Moderate |
| DEV:MSS | 0 | No concerns | Low risk | No concerns | No concerns | No concerns | No concerns | High |
| DEV:PLA | 0 | No concerns | Low risk | No concerns | No concerns | No concerns | No concerns | High |
| DEV:TBA | 0 | No concerns | Low risk | No concerns | No concerns | No concerns | No concerns | High |
| GFB:MSS | 0 | No concerns | Low risk | No concerns | No concerns | No concerns | No concerns | High |
| GFB:OXY | 0 | No concerns | Low risk | No concerns | Some concerns | No concerns | No concerns | Moderate |
| GFB:PLA | 0 | No concerns | Low risk | No concerns | No concerns | No concerns | No concerns | High |
| GFB:TBA | 0 | No concerns | Low risk | No concerns | No concerns | No concerns | No concerns | High |
| MSS:OXY | 0 | Some concerns | Low risk | No concerns | Some concerns | No concerns | No concerns | Low |
| MSS:TBA | 0 | No concerns | Low risk | No concerns | Some concerns | No concerns | No concerns | Moderate |
| OXY:PLA | 0 | No concerns | Low risk | No concerns | No concerns | No concerns | No concerns | High |
| OXY:TBA | 0 | Some concerns | Low risk | No concerns | Some concerns | No concerns | No concerns | Low |
| PLA:TBA | 0 | No concerns | Low risk | No concerns | No concerns | No concerns | No concerns | High |

Table S26 GRADE certainty of evidence for adverse events outcome in adult patients with diabetes mellitus and below-ankle diabetic foot ulcers

| Comparison | Number of studies | Within-study bias | Reporting bias | Indirectness | Imprecision | Heterogeneity | Incoherence | Confidence rating |
| --- | --- | --- | --- | --- | --- | --- | --- | --- |
| CAD:SOC | 1 | No concerns | Low risk | No concerns | Some concerns | No concerns | No concerns | Moderate |
| DEV:SOC | 5 | No concerns | Low risk | No concerns | Some concerns | No concerns | Some concerns | Low |
| GFB:SOC | 4 | No concerns | Low risk | No concerns | No concerns | Some concerns | No concerns | Moderate |
| MSS:SOC | 1 | Major concerns | Low risk | No concerns | Some concerns | No concerns | Some concerns | Very low |
| OXY:SOC | 5 | No concerns | Low risk | No concerns | No concerns | Some concerns | Some concerns | Low |
| CAD:DEV | 0 | No concerns | Low risk | No concerns | Some concerns | No concerns | No concerns | Moderate |
| CAD:GFB | 0 | No concerns | Low risk | No concerns | Some concerns | No concerns | No concerns | Moderate |
| CAD:MSS | 0 | No concerns | Low risk | No concerns | Some concerns | No concerns | Major concerns | Very low |
| CAD:OXY | 0 | No concerns | Low risk | No concerns | Some concerns | No concerns | Some concerns | Low |
| DEV:GFB | 0 | No concerns | Low risk | No concerns | Some concerns | No concerns | No concerns | Moderate |
| DEV:MSS | 0 | Major concerns | Low risk | No concerns | Some concerns | No concerns | Some concerns | Very low |
| DEV:OXY | 0 | No concerns | Low risk | No concerns | No concerns | Some concerns | No concerns | Moderate |
| GFB:MSS | 0 | Major concerns | Low risk | No concerns | Some concerns | No concerns | Some concerns | Very low |
| GFB:OXY | 0 | No concerns | Low risk | No concerns | Some concerns | No concerns | Major concerns | Very low |
| MSS:OXY | 0 | Major concerns | Low risk | No concerns | Some concerns | No concerns | Some concerns | Very low |

Table S27 GRADE certainty of evidence for amputation rate outcome in adult patients with diabetes mellitus and below-ankle diabetic foot ulcers

| **Therapy class** | **Main findings of the present meta-analysis** | **Certainty of evidence** | **2023 IWGDF/WGDF guideline recommendation** | **Recommendation strength / certainty of evidence** | **Overall interpretation** |
| --- | --- | --- | --- | --- | --- |
| OXY: oxygen-based therapies | Oxygen-based therapies showed potential benefit across several outcomes. In direct comparisons, OXY improved complete wound healing and was associated with lower amputation risk; in the network analysis, OXY ranked favorably for amputation-related outcomes. | Predominantly low to very low, varying by outcome | HBOT or topical oxygen therapy may be considered as adjunctive treatment when standard care fails, particularly in selected ischemic or neuroischemic DFUs. | Conditional recommendation; low-certainty evidence | The findings are broadly consistent with the guideline. OXY may be useful in selected patients, especially those with ischemic or neuroischemic ulcers, but the evidence does not support indiscriminate routine use. |
| DEV: device-based and physical wound therapies | DEV showed a strong signal for ulcer area reduction and also improved complete wound healing in direct comparisons. However, this category includes heterogeneous interventions such as NPWT, ESWT and ultrasound, and the evidence for healing time and durable clinical outcomes was less consistent. | Very low to moderate | NPWT may be considered for post-surgical diabetic foot wounds, but is not recommended for routine treatment of non-surgical DFUs. Other physical modalities are generally not recommended for routine ulcer healing. | NPWT for post-surgical wounds: conditional recommendation, low-certainty evidence; physical modalities: recommendation against routine use, low-certainty evidence | The meta-analysis suggests potential benefit for selected device-based interventions, but interpretation should distinguish post-surgical NPWT from non-surgical DFUs and other physical modalities. The broad DEV category should not be translated into a general recommendation for all device-based therapies. |
| GFB: growth factor and blood-derived therapies | GFB showed favorable effects on ulcer area reduction, complete wound healing, healing time and amputation in direct comparisons. Nevertheless, the category combines PRP/PRG, PDGF/becaplermin and rhEGF, and the consistency of evidence varied across outcomes and products. | Predominantly low to very low | Routine use of growth factors or autologous platelet-based products is not generally recommended, although selected autologous leukocyte-platelet fibrin products may be considered in specific circumstances. | Mostly conditional recommendation against routine use; low-certainty evidence | The present findings suggest biological plausibility and potential benefit, but remain insufficient to support routine use of all GFB therapies. Product-specific evidence should be interpreted separately rather than generalized to the whole class. |
| PLA: placental and perinatal tissue-derived products | PLA showed relatively consistent favorable effects, particularly for ulcer area reduction and complete wound healing, and ranked highly in several network outcomes. Some analyses also suggested a lower risk of adverse events, although safety reporting was heterogeneous. | Low to very low overall; certainty varied by outcome | Placental-derived products may be considered as adjunctive therapy after failure of standard care. | Conditional recommendation; low-certainty evidence | The findings are generally aligned with the guideline and support selective adjunctive use of placental-derived products. However, conclusions should remain cautious because products, preparation methods and application protocols differ substantially. |
| MSS: acellular matrix, skin substitutes and scaffold products | MSS showed benefit for complete wound healing and healing time, with relatively favorable direct-comparison results. However, this category includes diverse products such as acellular dermal matrices, collagen-based materials, fish skin grafts and other scaffold products. | Low to very low overall; some comparisons more precise | Routine use of most skin substitutes or biomaterials is not broadly recommended; selected products may be considered on a case-by-case basis when standard care fails. | Mostly conditional recommendations against routine use or selective use; low-certainty evidence | MSS appears promising, particularly for wound closure, but the results should not be generalized to all matrix or scaffold products. Product-level evidence, cost, availability and patient selection remain important. |
| CAD: cell-based and adipose-derived therapies | CAD showed a potential benefit for healing time and ranked favorably for this outcome, but evidence for complete wound healing, safety and amputation-related outcomes was limited and imprecise. | Very low to low, occasionally moderate depending on outcome | Routine use of cell-based therapies as adjunctive treatment is not recommended because of insufficient evidence. | Conditional recommendation against routine use; low-certainty evidence | The available evidence remains exploratory. CAD may be biologically promising, but current data are insufficient to support routine clinical use outside selected research or specialized settings. |
| TBA: topical pharmacologic and bioactive agents | TBA improved complete wound healing in direct comparisons and ranked moderately for adverse-event outcomes, but evidence was based on diverse agents with different mechanisms, including topical erythropoietin, esmolol, ON101 and other bioactive compounds. | Mostly low to very low | The guideline does not broadly recommend routine use of most topical pharmacologic agents for DFU healing, except for specific interventions supported by limited evidence in selected populations. | Generally no routine recommendation or conditional recommendation against routine use; low-certainty evidence | TBA should be interpreted as a heterogeneous exploratory category. Some agents may be promising, but class-level conclusions are premature and should not be used to justify routine adoption of all topical pharmacologic therapies. |
| NND: nanotechnology-based, natural product and bioactive dressing therapies | NND evidence was sparse. The category did not show consistent significant benefit in direct comparisons for ulcer area reduction or complete wound healing, and estimates were imprecise because few studies were available. | Very low | The guideline supports selected advanced dressings such as sucrose-octasulfate impregnated dressings in specific non-infected neuroischemic ulcers that fail standard care, but does not support routine use of many other natural, antimicrobial or experimental dressings. | Selected dressing: conditional recommendation; low-certainty evidence. Many other dressings: recommendation against routine use or insufficient evidence | The present findings are consistent with a cautious guideline position. Current evidence does not support routine use of NND as a broad category; any recommendation should be limited to specific products, indications and patient populations. |

Table S28 Abbreviations: CAD, cell-based and adipose-derived therapies; DEV, device-based and physical wound therapies; DFU, diabetic foot ulcer; GFB, growth factor and blood-derived therapies; HBOT, hyperbaric oxygen therapy; MSS, acellular matrix, skin substitute and scaffold products; NND, nanotechnology-based, natural product and bioactive dressing therapies; NPWT, negative pressure wound therapy; OXY, oxygen-based therapies; PLA, placental and perinatal tissue-derived products; SOC, standard of care; TBA, topical pharmacologic and bioactive agents.
